# A Novel Sandwich-Structured Phase Change Composite with Efficient Photothermal Conversion and Electromagnetic Interference Shielding Interface

**DOI:** 10.3390/ma17040961

**Published:** 2024-02-19

**Authors:** Jun Xu, Yuanyuan Li, Zhangxinyu Zhou, Xiaomin Cheng

**Affiliations:** 1 School of Materials Science and Engineering, Wuhan University of Technology, Wuhan 430070, China; xujun22@whut.edu.cn (J.X.); yyli@whut.edu.cn (Y.L.); zzxykeyan@whut.edu.cn (Z.Z.); 2 School of Electromechanical and Intelligent Manufacturing, Huanggang Normal University, Huanggang 438000, China

**Keywords:** organic composite phase change materials, thermal energy storage, multifunctional interfaces, photothermal conversion, electromagnetic interference shielding

## Abstract

Stability and multifunctionality greatly extend the applications of phase change materials (PCMs) for thermal storage and management. Herein, CuS and Fe_3_O_4_ nanoparticles were successfully loaded onto cotton-derived carbon to develop a multifunctional interface with efficient photothermal conversion and electromagnetic interference (EMI) shielding properties. 1,3:2,4-di-(3,4-dimethyl) benzylidene sorbitol (DMDBS) and expanded graphite (EG) formed an organic/inorganic three-dimensional network framework to encapsulate 1-octadecanol (OD) by self-assembly. Finally, multifunctional shape-stabilized PCMs (SSPCMs) with the sandwich structure were prepared by the hot-press process. Multifunctional SSPCMs with high load OD (91%) had favorable thermal storage density (200.6 J/g), thermal stability, and a relatively wider available temperature range with improved thermal conductivity to support the thermal storage and management realization. Furthermore, due to the synergistic enhancement of two nanoparticles and the construction of the carbon network with cotton carbon and EG, highly efficient photothermal conversion (94.4%) and EMI shielding (68.9 dB average, X-band) performance were achieved at about 3 mm thickness, which provided the possibility of the multifunctional integration of PCMs. Conclusively, this study provides new insights towards integrating solar energy utilization with the comprehensive protection of related electronics.

## 1. Introduction

Traditional fossil fuels are not renewable, and excessive use also brings about serious environmental damage [1]. Currently, climate deterioration has emerged as the most pressing problem facing the world [2]. As a result, scientists and researchers are seeking suitable renewable energy sources to combat climate deterioration worldwide. Solar energy is considered the most promising and important renewable energy source due to its abundant, free, and clean properties [3]. However, solar energy is intermittently in supply [4]. The continuous use of solar energy requires optimizing thermal energy storage (TES) and peak energy regulation. Latent heat storage is a way of TES that uses the endothermic and exothermic properties of phase change materials (PCMs) to realize energy storage with high energy storage density, slight temperature fluctuation, and good stability compared to other ways [5]. In particular, organic PCMs have great potential for intelligent wearable devices, energy-efficient construction, and battery thermal management [6,7]. Furthermore, organic PCMs can also be useful in solid slippery, antifouling, and liquid transport applications [8,9].

However, the drawbacks of poor thermal conductivity, liquid phase leakage, and supercooling limit the wider application of organic PCMs for thermal energy storage and management [10,11]. A lot of research has been conducted to alleviate these problems. Modified materials possessing excellent thermal conductivity and specific surface areas have been employed, including graphite derivatives [12], carbon nanotubes (CNTs) [13], and inorganic nanoparticles [14]. A variety of leakage-prevention methods have been devised to prepare shape-stabilized PCMs (SSPCMs), such as microencapsulation [15], porous adsorption [16], and hydrogel [17].

Conventional organic PCMs require passive heat absorption to raise the temperature above their melting point, lack active control, and are less efficient and more limited in practical applications [18]. Emerging photo-responsive materials bring new potential for organic PCMs due to their unique properties [19]. Zheng et al. [20] investigated a composite of graphene aerogel and copper foam encapsulating paraffin wax. The photothermal efficiency reached up to 97%, and the thermal conductivity was increased nine times at the mass fractions of 30% and 10% of the modified material, respectively. Nishad et al. [21] prepared a paraffin wax/graphite panel composite PCM by facile vacuum adsorption. The composites provided superior thermal and electrical conductivity, as well as a 76.5% photothermal conversion efficiency. Ye et al. [22] prepared Ti_3_C_2_-imported composite PCMs by carrying tetradecyl amine (TDA) in a hybrid aerogel, which exhibited photothermal conversion efficiencies up to 84.95% with a loading of more than 91.0 wt%. Kong et al. [23] coated polypyrrole on polydivinylbenzene nanotubes loaded with industrial paraffin waxes by rapid oxidative initiation, and the resulting composites had an 85.2% photothermal energy conversion effectiveness.

In addition, SSPCMs with high photothermal conversion efficiency have a widespread application prospect in fifth-generation (5G) communications, photothermal power generation, and power batteries for new energy vehicles, which are seriously affected by electromagnetic interference (EMI) in their operation [24]. Therefore, it is essential to simultaneously endow SSPCMs with excellent EMI shielding and photothermal conversion effectiveness. As a semiconductor material, CuS has a unique near-infrared localized surface plasmon resonance (LSPR) feature, which makes it an excellent photosensitive material [25]. Fe_3_O_4_ is one of the most promising materials for electromagnetic wave absorption due to its ferrite magnetism, moderate saturation magnetization strength, and strong spin polarization [26]. The current related studies generally consider single CuS [27,28] or Fe_3_O_4_ [29,30] nanomaterials to enhance the optical or magnetic properties of SSPCMs. And there are fewer reports on the co-enhancement of SSPCMs by CuS and Fe_3_O_4_. Therefore, we designed a multifunctional interface loaded with both CuS and Fe_3_O_4_ nanoparticles to synergistically enhance the photothermal conversion and electromagnetic interference shielding properties of SSPCMs. Compared with a single enhancement method, this approach not only makes SSPCMs multifunctional but also possesses a more excellent integrated performance, thus realizing the integration of efficient solar energy utilization with the thermal and electromagnetic protection of related devices.

**Figure 1:**
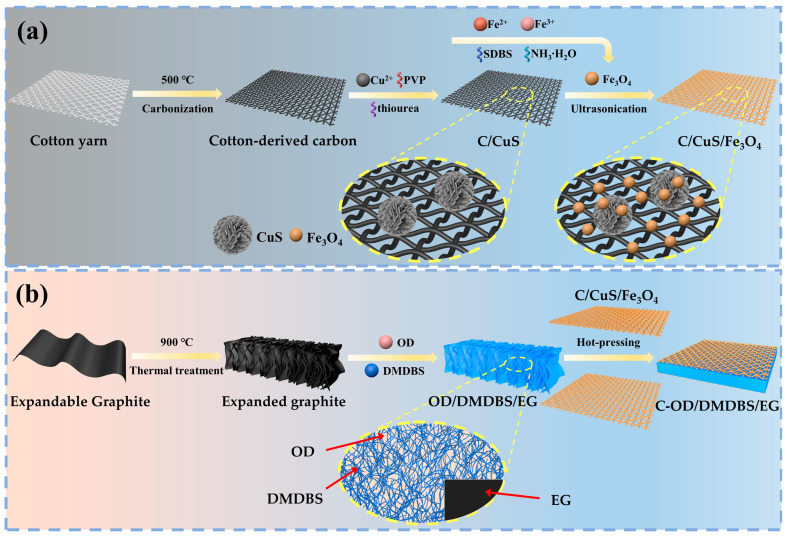
Schematic preparation of (**a**) C/CuS/Fe_3_O_4_ interface and (**b**) C-OD/DMDBS/EG composites.

In this work, we adopt a modular approach to prepare a novel SSPCM with a sandwich structure, which has efficient photothermal conversion and an EMI shielding interface. The schematic structure and preparation process are shown in Figure 1 and Figure S1.

CuS and Fe_3_O_4_ were synthesized by hydrothermal and co-precipitation methods and successfully loaded onto cotton-based carbon to obtain photothermal conversion and an EMI shielding interface as a multifunctional enhancement module. Subsequently, a low-molecular-weight organic gelator, 1,3:2,4-di-(3,4-dimethyl) benzylidene sorbitol (DMDBS), and expanded graphite (EG) formed a framework of an organic/inorganic three-dimensional network by self-assembly to encapsulate the PCM, 1-octadecanol (OD). Finally, multifunctional SSPCMs of a sandwich structure were prepared by assembling the multifunctional interface modules with SSPCMs using the hot-press process. Due to the synergistic effect of CuS and Fe_3_O_4_, as well as the 3D network constructed by organic/carbon materials, multifunctional SSPCMs are in the leading position in the comprehensive performance of thermal storage density, photothermal conversion, and EMI shielding, realizing the integration of solar energy utilization with the thermal and electromagnetic protection of related electronic devices and expanding applications in photothermal power generation, the integrated protection of electronics, and building energy efficiency. In addition, this modular preparation approach is facile and effective with fewer limitations, providing a novel strategy for the multifunctional integration of PCMs.

## 2. Materials and Methods

### 2.1. Materials

1-Octadecanol (C_18_H_38_O, OD, ≥99.0%), copper chloride (CuCl_2_, ≥98.0%), thiourea (CH_4_N_2_S, ≥99.0%), n-hexane (C_6_H_14_, ≥98.0%), ferrous chloride tetrahydrate (FeCl_2_·4H_2_O, ≥99.0%), and anhydrous ethanol (C_2_H_6_O, ≥99.7%) were procured from Shanghai Sinopharm Chemical Reagent Corporation in China. 1,3:2,4-di-(3,4-dimethyl) benzylidene sorbitol (C_24_H_30_O_6_, DMDBS, ≥99.0%), polyvinyl pyrrolidone (PVP, ≥98.0%), ferric chloride hexahydrate (FeCl_3_·6H_2_O, ≥99.0%), and sodium dodecylbenzene sulfonate (SDBS, ≥90.0%) were supplied by Shanghai Macklin Reagent Corporation in China. All chemicals and reagents required no additional treatment.

Expandable graphite (50 mesh) was provided by Qingdao Jinrilai Technology Corporation in China. Cotton yarn was obtained from Chongyang Steadfast Medical Supplies Corporation in China.

### 2.2. Synthesis of Multifunctional Interface Materials

Figure 1a illustrates the synthesis of a multifunctional interfacial material in three steps. Firstly, cotton-derived carbon materials were prepared (Figure S1a). Commercially purchased cotton yarn was selected as the precursor of cotton-derived carbon. The cotton yarn was first soaked in anhydrous ethanol and ultrasonically washed twice, dried at 60 °C for 1 h, and then carbonized under nitrogen protection by ramping up from room temperature to 500 °C (2 °C/min), held for 1 h, followed by natural cooling to obtain cotton-based carbon.

Secondly, the cotton-derived carbon/CuS (C/CuS) intermediate was synthesized in situ by a hydrothermal reaction (Figure S1b). A total of 50 mL of CuCl_2_ solution (0.01 g/mL) was prepared and stirred for 15 min. We then added 0.05 g of PVP and continued stirring for 15 min. Then, 20 mL of thiourea solution (0.1 g/mL) was dropped and mixed well until the solution was homogeneous. The mixture was then moved into a polytetrafluoroethylene (PTFE) reactor with cotton-derived carbon. The reactor was held airtight at 150 °C for 12 h and at 170 °C for 2 h, followed by natural cooling. The product was flushed with anhydrous ethanol and sufficiently desiccated for the C/CuS intermediate.

Finally, the cotton-derived carbon/CuS/Fe_3_O_4_ (C/CuS/Fe_3_O_4_) multifunctional interface materials were prepared (Figure S1c). Where Fe_3_O_4_ nanoparticles were prepared using the method in our previous work [29], the detail is presented in the S1.1. A total of 0.25 g of Fe_3_O_4_ nanoparticles was added to 40 mL of anhydrous ethanol and SDBS with ultrasonic dispersion for 30 min until uniformity was achieved. The C/CuS intermediate was submerged in the solution, ultrasonically dispersed, and continued for 5 min. The C-CuS intermediate adsorbed Fe_3_O_4_ nanoparticles through π-electrons and hydrophilic groups, while the highly electronegative SDBS adsorbed many Fe_3_O_4_ nanoparticles. The mixture was thoroughly desiccated for the C/CuS/Fe_3_O_4_ multifunctional interfacial materials.

### 2.3. Preparation of OD/DMDBS/EG Composites

OD/DMDBS/EG composites were prepared via vacuum melt adsorption as reported in our previous paper [31], as shown in Figure S1d. The preparation process is presented in the S1.2. Four samples with different DMDBS contents (wt%) were prepared and designated OD/1%DMDBS/6%EG, OD/3%DMDBS/6%EG, OD/5%DMDBS/6%EG, and OD/7%DMDBS/6%EG, respectively.

### 2.4. Preparation of SSPCMs with Multifunctional Interfaces

SSPCMs with the multifunctional interface of C/CuS/Fe_3_O_4_ featuring a sandwich structure were fabricated via the hot-press process (Figure S1e). At first, the bottom of a 12.5 mm diameter mold was lined with C/CuS/Fe_3_O_4_ and then filled with 320 ± 5 mg of OD/3%DMDBS/6%EG composites. Subsequently, C/CuS/Fe_3_O_4_ was spread on top, and the mold was pressed at 40 °C. After natural cooling, the product was removed from the mold to obtain C/CuS/Fe_3_O_4_-OD/3%DMDBS/6%EG (about 3 mm thickness). The hot-press process results in high proximity and increases the contact area between the components of the multifunctional SSPCM, which enhances the interparticle forces, such as van der Waals forces. In addition, the local temperature at some of the contact points can reach the melting point of OD, and oxygen-containing groups from both the melted OD and cotton-derived carbon formed hydrogen bonds with each other, further enhancing the adhesion between the C/CuS/Fe_3_O_4_ interface and OD/DMDBS/EG composites [32]. It was named C-OD/3%DMDBS/6%EG. Three additional composites were fabricated and designated as C-OD/1%DMDBS/6%EG, C-OD/5%DMDBS/6%EG, and C-OD/7%DMDBS/6%EG, respectively.

Furthermore, two samples without DMDBS were prepared as control samples and named C-OD/6%EG and C-OD/9%EG, respectively. Regarding the details of the “Characterization”, refer to the S1.3.

## 3. Results and Discussion

### 3.1. Structure and Composition

**Figure 2:**
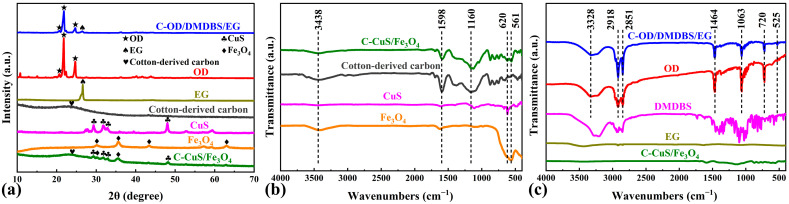
(**a**) XRD patterns and (**b**,**c**) FTIR spectra of C/CuS/Fe_3_O_4_, C-OD/DMDBS/EG, and individual components.

The XRD method was employed to characterize the crystal properties of multifunctional SSPCMs (Figure 2a). The prepared CuS has four obvious characteristic peaks (29.32°, 31.83°, 32.88°, and 47.92°) corresponding to the (102), (103), (006), and (110) planes of the standard diffraction peaks (ICDD No. 01-078-2122) [33], respectively.

Meanwhile, the prepared Fe_3_O_4_ has four obvious characteristic peaks (30.16°, 35.66°, 43.16°, and 62.79°) corresponding to the (220), (311), (400), and (440) planes of the standard diffraction peaks (ICDD No. 01-072-8149) [34], respectively. The results indicate that single-phase CuS [35] and Fe_3_O_4_ [36] were successfully prepared. In addition, as shown in Figure S2a, the relative diffracted intensity ratio of (110)/(103) in the CuS pattern is 1.43, which is significantly larger than the standard value (0.69) in the PDF card, suggesting that the CuS crystal orientation growth is preferred to the (110) plane [37]. However, no significant relative diffraction intensity changes are shown in the Fe_3_O_4_ pattern. Combined with Figure S2b, the diffraction peak pattern of the cotton-derived carbon shows a broad diffraction peak near 24.6° corresponding to amorphous carbon’s (002) plane, with no other obvious peaks, indicating that the cotton yarn was effectively carbonized. Since the pattern of C/CuS/Fe_3_O_4_ shows bulges in this location, cotton-derived carbon is identified as contained in the interface material [38]. Simultaneously, four CuS characteristic peaks and two Fe_3_O_4_ characteristic peaks appear on the pattern of C/CuS/Fe_3_O_4_. This indicates that CuS and Fe_3_O_4_ were successfully loaded on the cotton-derived carbon. It can also be found that the strong peaks located at 20.52°, 21.61°, and 24.50° in the C-OD/DMDBS/EG pattern correspond to the diffraction peaks of pure OD, while the strong peak at 26.61° corresponds to the diffraction peak of pure EG. Comparing Figure S2c, after the pure DMDBS powder was transformed into a 3D network structure by gelation, the initially sharp diffraction peaks observed in the C-OD/DMDBS/EG pattern appear rugged, probably due to the reduced crystallinity and lower content [39]. In addition, no significant diffraction peaks associated with C/CuS/Fe_3_O_4_ could be found in the C-OD/DMDBS/EG sample, which is attributed to poor content, resulting in weak diffraction peak intensity.

In the following paragraph, the chemical composition and structure of the C/CuS/Fe_3_O_4_ interface and C-OD/DMDBS/EG sample are investigated by FTIR analysis. The results for cotton-derived carbon, CuS, Fe_3_O_4,_ and C/CuS/Fe_3_O_4_ interface are shown in Figure 2b. The broad absorption peaks of the samples near 3438 cm^−1^ are attributed to the stretching vibration of O-H of absorbed water. Comparing the FTIR spectra of cotton yarn in Figure S3, after carbonization, the number of functional groups of cotton-derived carbon is significantly reduced, and the corresponding absorption peaks disappear or diminish. This indicates that a large amount of cellulose in cotton yarn was decomposed. For the interface materials, the absorption peak at 1598 cm^−1^ is attributed to the C=C skeleton stretching vibration in cotton-derived carbon. The absorption peak around 1160 cm^−1^ is associated with a substantial decomposition of cellulose (asymmetric stretching vibration for C-O in C-O-C) [40]. The Cu-S (620 cm^−1^) and Fe-O (561 cm^−1^) contraction vibration peaks are related to CuS [41] and Fe_3_O_4_ [42] in the C/CuS/Fe_3_O_4_ interface, respectively. Together with the XRD results, these indicate that CuS and Fe_3_O_4_ are successfully loaded on cotton-derived carbon. The spectrum of C-OD/DMDBS/EG retained the curve of pure OD almost completely, as shown in Figure 2c. The main absorption bands are corresponding to C-C skeleton stretching vibrations (525 cm^−1^), C-O stretching vibrations (1063 cm^−1^), C-H stretching vibrations in -CH_2_ groups (2851 cm^−1^ and 2918 cm^−1^), and the bending and stretching vibrations of O-H (720 cm^−1^, 1464 cm^−1^, and 3328 cm^−1^) [43]. Therefore, there is no chemical change in the OD in the composites, and only physical forces exist between it and the modified one, which ensures an excellent thermal storage capacity. In addition, similar to the XRD analysis, most of the absorption peaks of pure DMDBS disappear or diminish after gelation. The low content and weak absorption vibrational peaks of EG and C/CuS/Fe_3_O_4_ result in their characteristic peaks being inconspicuous in the C-OD/DMDBS/EG spectra. The successful preparation of SSPCM with the multifunctional interface is demonstrated based on XRD and FTIR analysis.

### 3.2. Microscopic Morphology

**Figure 3:**
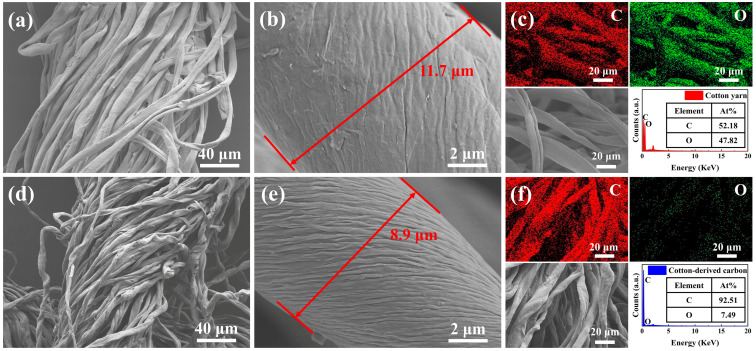
SEM micrographs of (**a**,**b**) cotton yarn and (**d**,**e**) cotton-derived carbon; EDX and elemental distribution images of (**c**) cotton yarn and (**f**) cotton-derived carbon.

The microscopic morphology of the multifunctional interface and OD/DMDBS/EG composites was investigated by SEM, TEM, and EDX. Figure 3 shows the microscopic morphology and elemental distribution of cotton yarn and cotton-derived carbon.

In Figure 3a,b,d,e and Figure S4, the cotton yarn changed from the original white to black after heat treatment at 500 °C and underwent significant shrinkage. The edge length of the cotton-derived carbon is about 77% of the original size, and the single fiber is about 76% of the original size. Meanwhile, the fiber surface of the cotton-derived carbon is significantly roughened, facilitating nanoparticle loading. In the elemental mapping image of Figure 3c,f, the contents of C and O in the cotton yarn are relatively close, with an atomic number ratio of about 1.09. After carbonization, the O content is significantly lower, with an atomic number ratio of C to O of about 12.35. This is conducive to enhancing the thermal and electrical conductivity of cotton-derived carbon.

**Figure 4:**
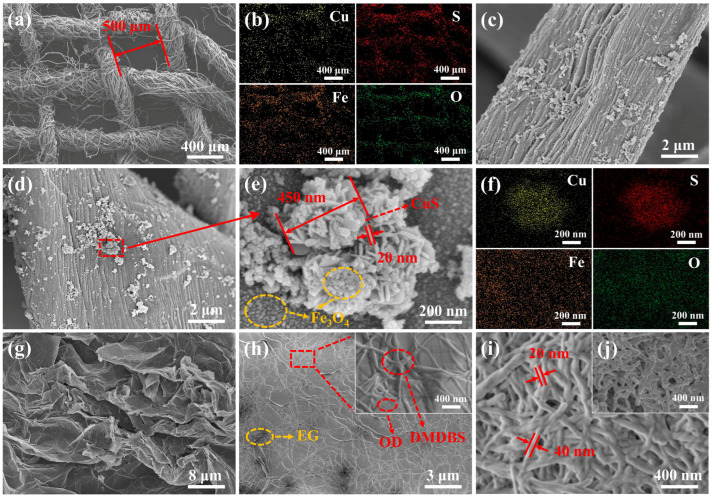
SEM micrographs for (**a**,**c**,**d**,**e**) C/CuS/Fe_3_O_4_ interface, (**g**) EG, (**h**) OD/DMDBS/EG composites, and xerogel of (**i**) OD/3%DMDBS/6%EG and (**j**) OD/1%DMDBS/6%EG; elemental mapping images of (**b**,**f**) C/CuS/Fe_3_O_4_ interface.

SEM and EDX patterns of the multifunctional interface and OD/DMDBS/EG samples are shown in Figure 4. As shown in Figure 4a, the side length of the holes formed by the warp and weft lines of cotton-derived carbon is about 500 μm.

In the elemental mapping image of Figure 4b, the elements Cu, S, Fe, and O can be detected in the multifunctional interface and uniformly distributed on the cotton-derived carbon. As shown in Figure 4c–f, CuS and Fe_3_O_4_ nanoparticles are uniformly loaded on the cotton-derived carbon. The microscopic morphology of the prepared CuS is a three-dimensional multistage microsphere structure formed by the self-assembly and cross-stacking of two-dimensional nanoflake layers with a thickness of about 20 nm. The diameter of the microsphere structure is about 450 nm. Numerous open pores exist on the surface of the microspheres, which promotes the loading of Fe_3_O_4_. In addition, the prepared Fe_3_O_4_ micromorphology is in the form of nanoparticles averaging about 10 nm in diameter, loaded on cotton-derived carbon and partially on CuS microspheres. This demonstrates the successful preparation of interface materials loaded with CuS and Fe_3_O_4_ nanoparticles. The microscopic morphology of EG, xerogel, and OD/DMDBS/EG is shown in Figure 4g–j. Xerogel was prepared by washing OD/DMDBS/EG thoroughly with n-hexane, filtering out the pure OD, and then drying under vacuum for 24 h. As seen in Figure 4g, massive interlocking graphite layers appear after EG expansion, forming extensive micropores that can be used as a framework for support and adsorption. As shown in Figure 4h, many organic composites are distributed on the surface of EG and in the micropores, and OD is dispersed into a fibrous network of DMDBS. OD/DMDBS/EG relies on the organic/inorganic 3D network framework of EG and DMDBS to package OD. Figure 4i shows the xerogel of OD/DMDBS/EG with 3% of DMDBS addition. After removing OD, EG is covered with a large amount of xerogel, a compact 3D fibrous network (fiber diameter, approximately 30 nm) created from the self-assembly by DMDBS. In Figure 4j, the content of DMDBS in OD/DMDBS/EG is 1%. It can be observed that DMDBS does not form a complete and dense 3D network, and there are prominent discontinuous fibers with unsatisfactory leakage prevention. In addition, to further discuss the contribution of DMDBS to the shape stability of the composites, the gel–sol transition temperature of the OD/DMDBS gelatinous composites was tested by the falling ball method [44]. The test method is presented in the SI, and the results are shown in Figure S5. It can be observed that the gel–sol transition temperatures of the OD/DMDBS composites are all obviously higher than the melting temperature of OD. This indicates that DMDBS enables the composites to maintain a stable shape at temperatures above the melting temperature of OD. Moreover, the temperature difference between the gel–sol transition temperatures of the composites with 3% DMDBS and those with 1% DMDBS is more than 60 °C, indicating better shape stability. This is similar to the SEM results.

**Figure 5:**
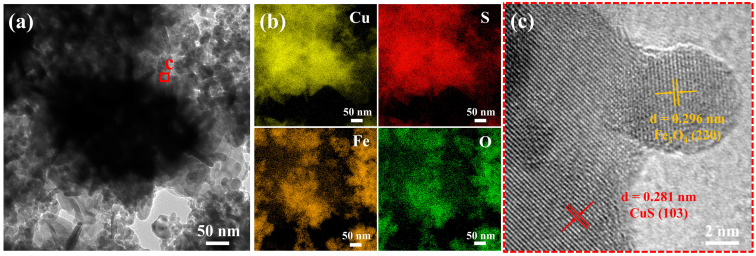
(**a**) TEM micrograph of CuS/Fe_3_O_4_ nanoparticles; corresponding (**b**) element mapping and (**c**) HRTEM images.

The TEM micrograph, elemental distribution, and HRTEM image of CuS/Fe_3_O_4_ nanoparticles are presented in Figure 5.

In Figure 5a,b, similar to the SEM images, Fe_3_O_4_ is evenly dispersed on and around the CuS microspheres. Figure 5c illustrates the HRTEM results for the labeled positions. Lattice face sets with a spacing of 0.281 nm and 0.296 nm can be found, corresponding to the (103) face of CuS and the (220) face of Fe_3_O_4_, respectively. These further indicate the successful preparation of multifunctional interfaces loaded with CuS and Fe_3_O_4_.

### 3.3. Thermal Storage Properties and Cycling Stability

**Figure 6:**
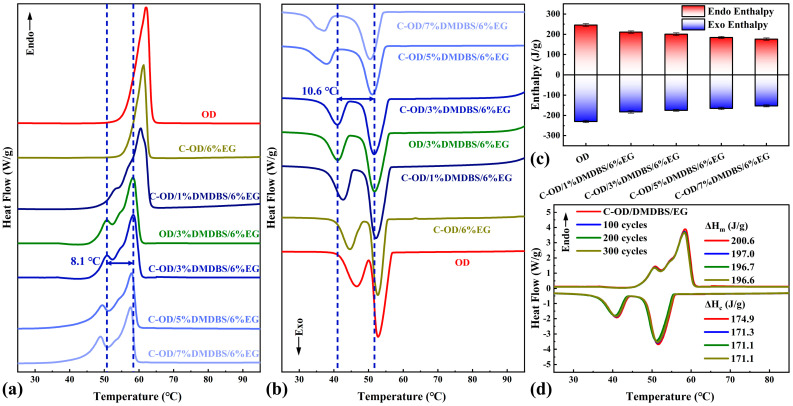
DSC (**a**) melting curves, (**b**) crystallization curves, and (**c**) enthalpies of phase transitions for various samples; (**d**) DSC cycling results for C-OD/3%DMDBS/6%EG.

The latent heat storage performance of composite PCMs is a key indicator for evaluating their application prospects. The phase transition temperatures and enthalpies of C-OD/DMDBS/EG with different compositions were measured via DSC analysis, and the effects of the components in C-OD/DMDBS/EG on their energy storage properties were investigated. Moreover, pure OD, C-OD/6%EG, and OD/3%DMDBS/6%EG were prepared as control samples. Enthalpies of melting and crystallization (ΔH_m_ and ΔH_c_) with corresponding peak temperatures (T_m_ and T_c_) for various samples are presented in Table 1. Furthermore, the degree of crystallinity (χ_c_) is obtained from the following Equation (1) [45]:

(1) $$\chi_{c}=\frac{{\Delta H}_{m}}{{\Delta H}_{mth}}$$

where ΔH_mth_ is the theoretical melting enthalpy. In Figure 6a,b, the endothermic peak of the pure OD is a singular peak, while the exothermic peak appears as two different peaks.

This is due to the two-step phase change process of OD, which includes solid–solid and solid–liquid phase transition [46]. Comparing OD/6% EG and pure OD, the melting and crystallization peak temperatures are very close, and the endothermic peaks are single, whereas the samples containing DMDBS show obvious peak separation and an overall shift towards lower temperatures. This is due to the nanoconfinement effect of organic/inorganic 3D network frameworks [47,48]. The melting peak temperature of C-OD/3%DMDBS/6%EG is 58.3 °C, with a peak temperature difference from the solid–solid phase transition of about 8.1 °C. This meant that C-OD/3%DMDBS/6%EG could phase change even at temperatures below 50 °C, giving an extended range of usable temperatures. Similarly, the crystallization peak temperature of C-OD/3%DMDBS/6%EG was 51.6 °C, which is about 10.6 °C different from the peak temperature of the solid–solid phase transition.

As shown in Figure 6c, the phase change enthalpies of the composites decrease gradually as there is an increase in the modified materials. The main reason for this is that none of the modification materials undergo phase transition during the OD phase transition. In addition, the crystallinity of the composites gradually decreases, and the crystallinity of C-OD/7%DMDBS/6%EG was only 84.4%. The reason for this may be that part of the molecular chain of OD is constrained by the organic/inorganic 3D network framework, and the phase transition was hindered [49]. Furthermore, according to our previous work, the organic/inorganic 3D network framework also alters the mechanism of the first step of the phase transition reaction model while significantly changing the activation energies of the two steps, in which DMDBS plays a dominant role [50]. In spite of this, the phase transition enthalpies of C-OD/3%DMDBS/6%EG still reach 200.6 J/g and 174.9 J/g, with favorable thermal storage performance. In addition, when comparing the results for OD/3%DMDBS/6%EG, the introduction of the multifunctional interface had no significant effect on the DSC, only resulting in a slight decrease in the enthalpy value of C-OD/3%DMDBS/6%EG due to the absence of a phase change in the interfacial material. This modular preparation method results in multifunctional interfaces not causing additional interference with the phase changes in the composites.

To estimate the thermal cycling stability of the multifunctional SSPCMs, the C-OD/3%DMDBS/6%EG sample was subjected to 300 thermal cycles. As shown in Figure 6, the DSC curves of the multifunctional SSPCM still basically overlap even after 300 thermal cycles, and the phase change enthalpies only slightly decrease. The ΔH_m_ and ΔH_c_ are 196.6 J/g and 171.1 J/g, just 2.0% and 2.2% lower than the initial values. The cycling test results show that the multifunctional SSPCMs have excellent stability, and the organic/inorganic 3D network framework provides reliable protection for the adsorbed OD.

### 3.4. Thermal and Shape Stability

**Figure 7:**
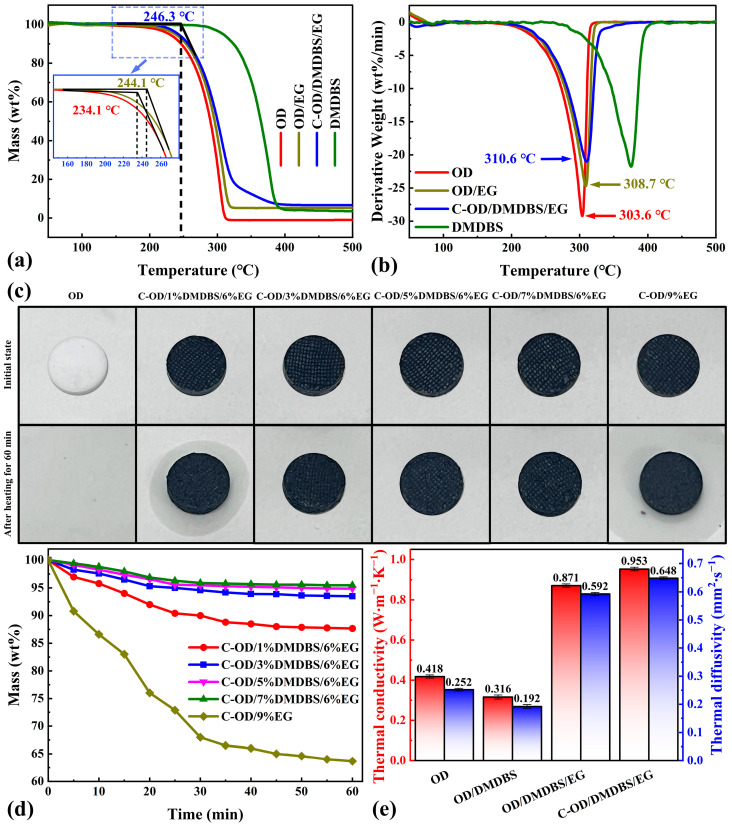
(**a**,**b**) TG-DTG curves of OD, DMDBS, OD/6%EG, and C-OD/3%DMDBS/6%EG; (**c**) digital photographs of OD, C-OD/1%DMDBS/6%EG, C-OD/3%DMDBS/6%EG, C-OD/5%DMDBS/6%EG, C-OD/7%DMDBS/6%EG, and C-OD/9%EG heated for 60 min to 80 °C; (**d**) the relative remaining mass of the different samples; (**e**) thermal diffusivity and thermal conductivity of OD, OD/3%DMDBS, OD/3%DMDBS/6%EG, and C-OD/DMDBS/EG.

Figure 7a,b show the TG and DTG results for the multifunctional SSPCM and components.

Pure OD shows a one-step thermal degradation process with completion at around 313.1 °C (Figure 7a). However, C-OD/3%DMDBS/6%EG shows a two-step thermal degradation process, with the first step being mainly the thermal degradation of OD. The second step starts at about 325 °C, and the thermal degradation slows down significantly, which should be due to the start of the thermal degradation of DMDBS. Finally, rapid thermal degradation is completed at about 390.6 °C. Additionally, a slight mass loss is observed already before 200 °C for pure OD, but almost no mass loss occurs for C-OD/3%DMDBS/6%EG. The main reason for this is the confinement of OD within the 3D network of DMDBS. The extrapolation of TG curves at the beginning of rapid thermal degradation yields an onset decomposition temperature for C-OD/3%DMDBS/6%EG (246.3 °C) that is close to that for OD/6%EG (244.1 °C) and 12.2 °C higher than that for OD (234.1 °C). At this time, the organic 3D network has melted, and the main reason for increasing the onset decomposition temperature of the composite PCMs is that the porous framework of EG hinders the rapid decomposition of the OD. The DTG curves in Figure 7b show that the maximum decomposition rate of the C-OD/DMDBS/EG sample corresponds to a temperature 7.0 °C higher than that for OD. At this time, EG still plays a significant role. This indicates that the organic/inorganic 3D network framework effectively prevents the thermal decomposition of the OD and improves thermal stability.

A high-temperature thermal shock experiment tested the shape stability of multifunctional SSPCMs. Four multifunctional SSPCMs with different DMDBS contents were investigated, with pure OD and C-OD/9%EG selected as control samples. Various samples of the same mass were compressed as circular flakes (12.5 mm in diameter) and laid on the heated table at a temperature of 80 °C, with filter paper underneath. The samples were removed every 5 min and weighed to calculate the change in mass. Figure 7c shows digital photographs of the samples before and after 60 min of heating. Pure OD completely melts into liquid after 10 min and is absorbed by the filter paper. The C-OD/1% DMDBS/6% EG sample partially leaks after 60 min of heating but keeps its original shape. As the content of DMDBS increases, the samples retain their original shape, and no significant leakage is observed. As a control sample, although C-OD/9%EG is added with the same content of modified material as C-OD/3%DMDBS/6%EG, more obvious leakage is observed than C-OD/1%DMDBS/6%EG. Figure 7d shows the curves of the masses for various samples with heating time. After 60 min of heating, the remaining mass of C-OD/9%EG is only 63.68% from the initial state, and that of C-OD/1%DMDBS/6%EG is 87.66%. With the increase in the DMDBS percentage, the remaining mass of C-OD/3%DMDBS/6%EG reaches 93.49% of the initial state, showing excellent antileakage performance. After 60 min of heating, the remaining mass of C-OD/9%EG is only 63.68% of the initial state, that of C-OD/1%DMDBS/6%EG is 87.66%, while C-OD/3% DMDBS/6%EG reaches 93.49%. These demonstrate that adding a small amount of DMDBS can significantly enhance the leakage prevention performance of composites, and the organic/inorganic 3D network framework ensures the shape stability and antileakage performance of the multifunctional SSPCMs.

### 3.5. Thermal Conductivity and Conditioning

OD, as an organic material, has a low thermal conductivity and diffusivity of 0.418 W∙m^−1^∙K^−1^ and 0.252 mm^2^∙s^−1^, respectively, whereas OD/3%DMDBS shows reductions of 24.4% and 31.3% compared to OD (Figure 7e). After adding EG, the OD/3%DMDBS/6%EG results are significantly improved by 108.4% and 134.9% compared to OD. This is attributed to the porous framework of the EG providing 3D heat conduction channels for organic composites. In addition, the thermal conductivity and diffusivity of C-OD/3%DMDBS/6%EG are further improved by the introduction of C/CuS/Fe_3_O_4_ interfacial materials, which are 0.953 W∙m^−1^∙K^−1^ and 0.648 mm^2^∙s^−1^, respectively, representing an increase of 128.0% and 157.1% compared with OD. This may be due to interfacial materials with high thermal conductivity complementing the discontinuous fast heat transfer channels on the OD/DMDBS/EG surfaces.

**Figure 8:**
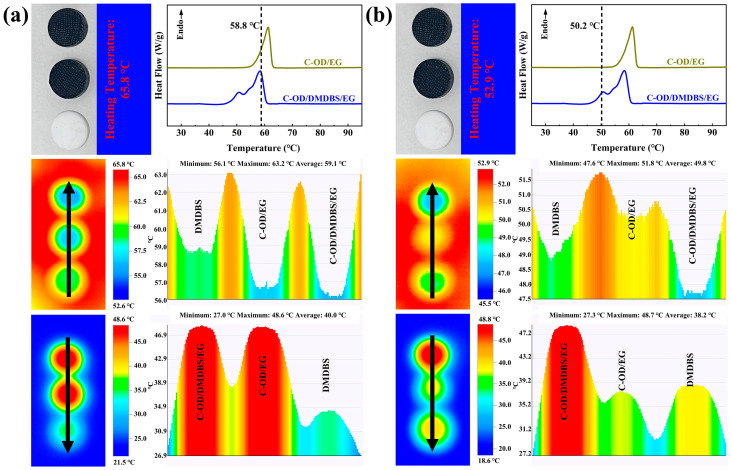
Infrared thermal maps of C-OD/3% DMDBS/6% EG, C-OD/6% EG, and pure OD upon heating and cooling at heating temperatures of (**a**) 65.8 °C and (**b**) 52.9 °C.

To investigate the thermoregulatory properties of the multifunctional SSPCMs, an infrared thermography camera was used for evaluation. Pure DMDBS was selected as the control sample without the addition of PCM, and C-OD/6%EG was selected as the control sample without the addition of the gel factor. The same mass of C-OD/3% DMDBS/6%EG, C-OD/6%EG, and pure OD samples was compressed as circular flakes (12.5 mm in diameter) and laid on the heated table from top to bottom. As shown in Figure 8a, when the temperature of the heating table is 65.8 °C, pure DMDBS rapidly rises to 58.8 °C for 30 s upon heating.

Heating continues until the temperatures of the three samples are almost the same. Then, after cooling at room temperature, the temperature of pure DMDBS rapidly cools to 34.3 °C. Meanwhile, the C-OD/3%DMDBS/6%EG and C-OD/6%EG samples show a significant hysteresis during heating and cooling. This is because the pure DMDBS does not undergo phase change. In contrast, the composite PCMs, when adding OD, undergo phase transition processes, which absorb and release large amounts of heat, and the temperatures do not increase or decrease rapidly, playing a thermal buffer role. As shown in Figure 8b, when the heating table temperature is 52.9 °C, the temperature of C-OD/6%EG increases rapidly to 50.2 °C after heating for 25 s without starting the phase transition. By now, the temperature of pure DMDBS is lower at 48.9 °C. This is because EG and interfacial materials enhance thermal conduction. However, the temperature of C-OD/3%DMDBS/6%EG is only 47.7 °C, which indicates that C-OD/3%DMDBS/6%EG has already started the solid–solid phase transition, which is in agreement with the DSC results. Introducing the gel factor DMDBS increases the temperature range of phase change, allowing it to undergo phase transition energy storage and temperature regulation at lower temperatures. Heating continues until the temperatures of the three samples are essentially the same, and then they cool at room temperature. Similarly, C-OD/6%EG rapidly cools to 37.4 °C, and the temperature of the pure DMDBS sample is slightly higher at 38.7 °C. Meanwhile, the temperature of C-OD/3% DMDBS/6%EG is maintained at 48.8 °C. The release of the latent heat of the phase transition plays a key role. This indicates that the multifunctional SSPCMs have superior thermal regulation and a wider range of available temperatures than PCMs without added DMDBS.℃

### 3.6. Photothermal Conversion

**Figure 9:**
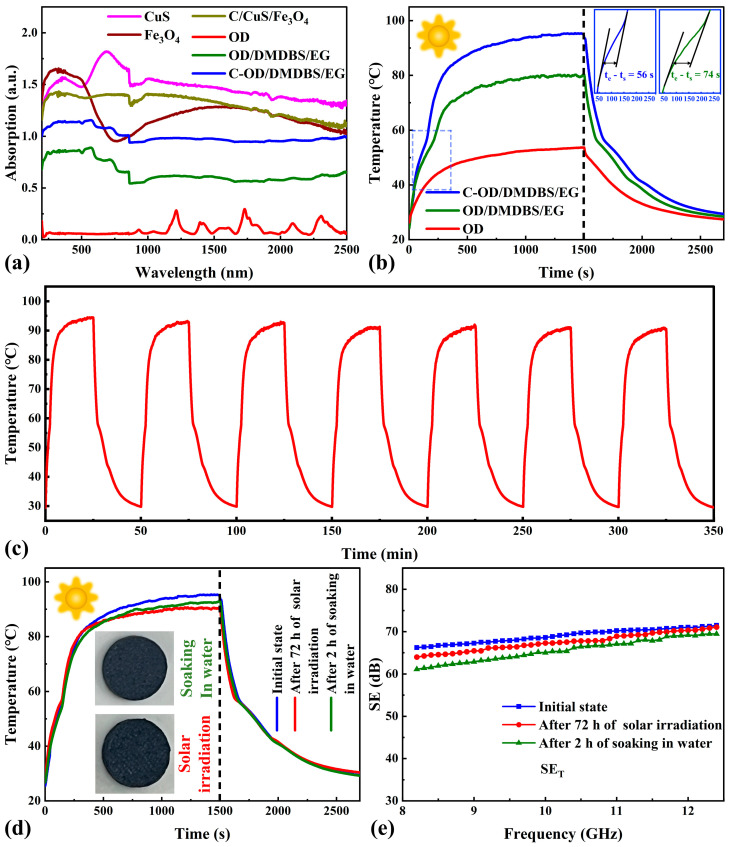
(**a**) UV-VIS-NIR absorbance spectrum of C-OD/3%DMDBS/6%EG and components; (**b**) temperature profiles under illumination of OD, OD/3%DMDBS/6%EG, and C-OD/3%DMDBS/6%EG; (**c**) temperature profiles of C-OD/3%DMDBS/6%EG at 7 complete cycles; (**d**) temperature profiles under illumination and (**e**) EMI SE_T_ of C-OD/3%DMDBS/6%EG after 72 h solar irradiation or 2 h soaking in water.

The ultraviolet–visible–near infrared (UV-Vis-NIR) absorptance spectrum for pure OD, nanoparticles, interface material, OD/3%DMDBS/6%EG, and C-OD/3%DMDBS/6%EG is shown in Figure 9a.

Pure OD shows almost zero absorption in the visible region and deficient absorption throughout the wavelength band, whereas OD/3%DMDBS/6%EG shows an enhanced absorption intensity throughout the spectral range compared to pure OD. This is due to a blackbody-like property and a large number of micropores in EG [51]. The C/CuS/Fe_3_O_4_ interface material and the C-OD/DMDBS/EG with a multifunctional interface exhibit significantly higher light absorption throughout the UV-Vis-NIR range, showing excellent absorption properties. The enhancement of absorbance peaks near 692 nm and 1048 nm should correlate with CuS. These are attributed to the LSPR phenomenon of CuS [52] and Fe_3_O_4_ [53] nanoparticles. Introducing multifunctional interfaces can effectively enhance the light absorption performance of OD/3%DMDBS/6%EG and more efficiently convert the photos to heat, thus heating the phase change material OD and realizing photothermal energy storage.

To assess the photothermal conversion efficiency, one specialized test system was built, as shown in Figure S6. Three samples of pure OD, OD/3%DMDBS/6%EG, and C-OD/3%DMDBS/6%EG were selected, weighed with a 320 ± 5 mg mass, and pressed into 12.5 mm diameter circular flakes for testing. Figure 9b illustrates the temperature profiles of the three samples with irradiation time under simulated solar irradiation with an 850 mW/cm^2^ intensity. These three samples were heated up at room temperature. The pure OD temperature increases relatively slowly as the illumination increases, reaching only 53.6 °C after 15 min without phase transition. Therefore, no melting or crystallization plateau is observed in its curve. In contrast, OD/3%DMDBS/6%EG shows a faster rate of temperature rise. At 45.6 °C, a platform occurs, with a significant decrease in the rate of temperature rise as OD melts. After complete melting, the temperature continues to rise and eventually reaches about 80.3 °C. Subsequent to cooling, a platform resembling the endothermic process occurs due to the crystallization of the OD. For C-OD/3%DMDBS/6%EG, the fastest rate of temperature rise is observed, and after completing the phase transition process, the rapid temperature rise continues to reach a final temperature of about 95.4 °C. This may be due to the excellent light absorption at the C/CuS/Fe_3_O_4_ interface, further enhancing the photothermal conversion performance of OD/3%DMDBS/6%EG. These findings are in general agreement with the results of Figure 9a.

The photothermal conversion efficiencies (η) for C-OD/3%DMDBS/6%EG and OD/3%DMDBS/6%EG are derived from Equation (2), as follows [54]:

(2) $$\eta =\frac{{m\Delta H}_{m}}{PS(t_{e}-t_{s})}$$

where m is the total mass of OD/3%DMDBS/6%EG (319.4 mg) and C-OD/3%DMDBS/6%EG (322.1 mg); ΔHm is the enthalpy of OD/3%DMDBS/6%EG (204.8 J/g) and C-OD/3%DMDBS/6%EG (200.6 J/g); P is the radiation intensity; and t_s_ and t_e_ are the starting and ending times of the melting processes. As shown in Figure 9b, the tangent method can obtain ts and te. The photothermal conversion efficiencies of OD/3%DMDBS/6%EG and C-OD/3%DMDBS/6%EG samples are calculated as 72.8% and 94.4%, respectively. Introducing a C/CuS/Fe_3_O_4_ interface improves the photothermal conversion efficiency of OD/3% DMDBS/6% EG by 21.6%. The results indicate that incorporating the multifunctional interface improves the photothermal conversion property of OD/DMDBS/EG remarkably. The photothermal conversion cycling curves for C-OD/3%DMDBS/6%EG are illustrated in Figure 9c. C-OD/3%DMDBS/6%EG maintains similar temperature change curves over seven consecutive solar irradiation and natural cooling cycles. Corresponding to the thermal cycling results of DSC, it is shown that the investigated multifunctional SSPCMs have superior photothermal cycling and thermal stability properties. To examine the stability of multifunctional SSPCM under the high-temperature environment of prolonged solar irradiation, C-OD/3%DMDBS/6%EG was continuously exposed to simulated solar irradiation at a density of 850 mW/cm^2^ for 72 h, and the sample temperature was kept above 90 °C. Afterwards, the digital photograph taken in Figure 9d represents that OD/3%DMDBS/6%EG still maintains a stable macroscopic shape. The photothermal conversion test result in Figure 9d indicates that the sample supports a high photothermal conversion performance, with the maximum temperature only reducing by 4.1% compared to the initial state. To evaluate the stability of the multifunctional SSPCMs in a humid environment, C-OD/3%DMDBS/6%EG was soaked in deionized water for 2 h and then removed for testing. As shown in the digital photograph of Figure 9d, the shape of the sample is stable. As shown in the photothermal conversion test results in Figure 9d, the dried sample exhibits a similar photothermal conversion performance to the initial state, with only a 1.4% decrease in maximum temperature.

### 3.7. Electromagnetic Shielding Performance

**Figure 10:**
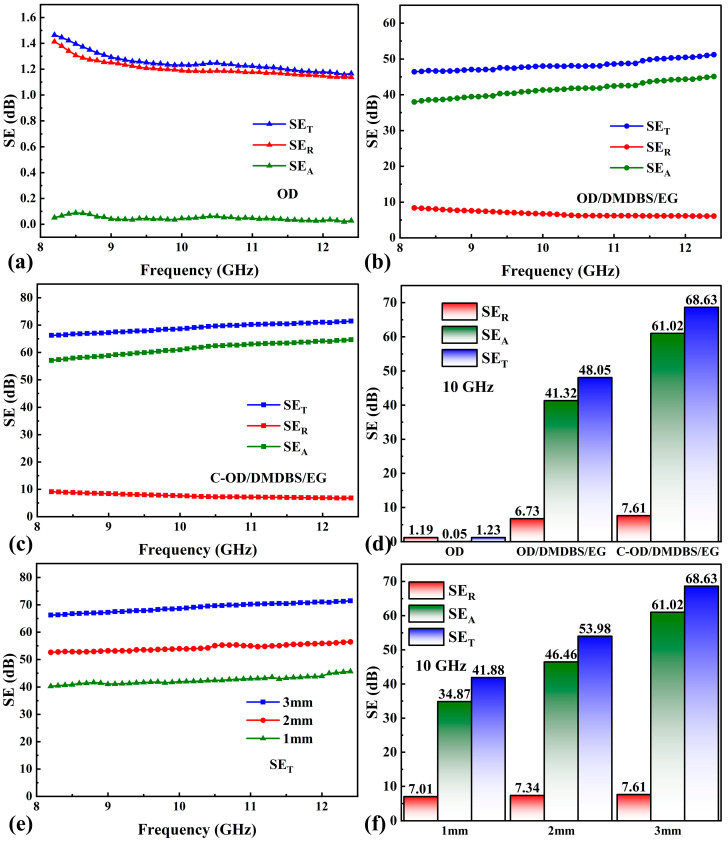
The EMI SE of (**a**) OD, (**b**) OD/3%DMDBS/6%EG, (**c**) C-OD/3%DMDBS/6%EG; (**d**) SE_T_, SE_R_, and SE_A_ of different samples at a frequency of 10 GHz; (**e**) SE_T_ of C-OD/3%DMDBS/6%EG with different thickness; (**f**) SE_T_, SE_R_, and SE_A_ of C-OD/3%DMDBS/6%EG with different thickness at a frequency of 10 GHz.

Shielding effectiveness (SE) is a critical metric in assessing resistance to EMI. The EMI SE data of pure OD, OD/3%DMDBS/6%EG, and C-OD/3%DMDBS/6%EG samples from 8.2 to 12.4 GHz (X-band) are presented below in Figure 10a–d.

As shown in Figure 10a, the pure OD samples exhibited extremely low SE over the entire frequency range. Electrical conductivity directly affects the EMI shielding capability [55]. Therefore, organic insulating materials could not be used directly for EMI. As shown in Figure 10b, with the addition of EG, the SE of the OD/3%DMDBS/6%EG sample significantly increases compared to the pure OD, reaching an average value of 48.8 dB. This is attributed to a dense 3D conductive network of EG in the sample obtained by high-pressure pressing, which results in a higher electrical conductivity (11.3 S/cm) for the OD/3%DMDBS/6%EG. This exhibits a stronger interaction with incident radiation, thus improving the shielding effectiveness. The SE of C-OD/3%DMDBS/6%EG is further improved by introducing the C/CuS/Fe_3_O_4_ interface, with an average value of up to 68.9 dB (Figure 10c). The main reason for this is the strongly magnetic Fe_3_O_4_ [56]. Additionally, CuS [57] contains many mobile charge carriers, which enhances electromagnetic shielding further. As shown in Figure 10d, the reflection, absorption, and summed EMI SE for the samples at 10 GHz are compared. The SE_A_ for both C-OD/3%DMDBS/6%EG and OD/3%DMDBS/6%EG is significantly larger than the SE_R_, indicating that most of the incident electromagnetic waves are absorbed rather than reflected. This characteristic dramatically decreases the reflections of EM waves, thus actively reducing the contamination of EM radiation [58]. To investigate the correlation between the EMI shielding performance and the thickness of the multifunctional SSPCMs, C-OD/3%DMDBS/6%EG samples with thicknesses of 1 mm and 2 mm, respectively, were additionally prepared by decreasing the corresponding ratio of OD/3%DMDBS/6%EG. The EMI SE of C-OD/3%DMDBS/6%EG with different thicknesses and its reflection, absorption, and total SE at 10 GHz are shown in Figure 10e,f. It can be seen that the EMI SE is positively correlated with the thickness of the C-OD/3%DMDBS/6%EG, which is attributed to the reflection and multiple reflection increases. In addition, the C-OD/3%DMDBS/6%EG sample with a thickness of 1 mm still exhibits a decent SE of 43.75 dB on average. The effect of the high-temperature environment of prolonged solar irradiation on the EMI shielding performance is also a critical issue in practical applications. As shown in Figure 9e, after 72 h of exposure to the high-temperature (above 90 °C) environment of solar irradiation at a density of 850 mW/cm^2^, the average EMI SE of OD/3%DMDBS/6%EG decreases by only 1.4 dB. This indicates that even in a high-temperature environment with prolonged solar irradiation, the multifunctional SSPCM can still maintain a high EMI shielding performance. As shown in Figure 9e, the average EMI SE of OD/3%DMDBS/6%EG decreases by only 3.6 dB after being immersed in water for 2 h, indicating that OD/3%DMDBS/6%EG can shield electromagnetic interference even in a humid environment. In addition, recent similar studies are summarized in Table 2, and the results indicate that the C-OD/3%DMDBS/6%EG in this study has a leading advantage when considering the three important indexes of latent heat storage density, photothermal conversion efficiency, and EMI SE.

**Figure 11:**
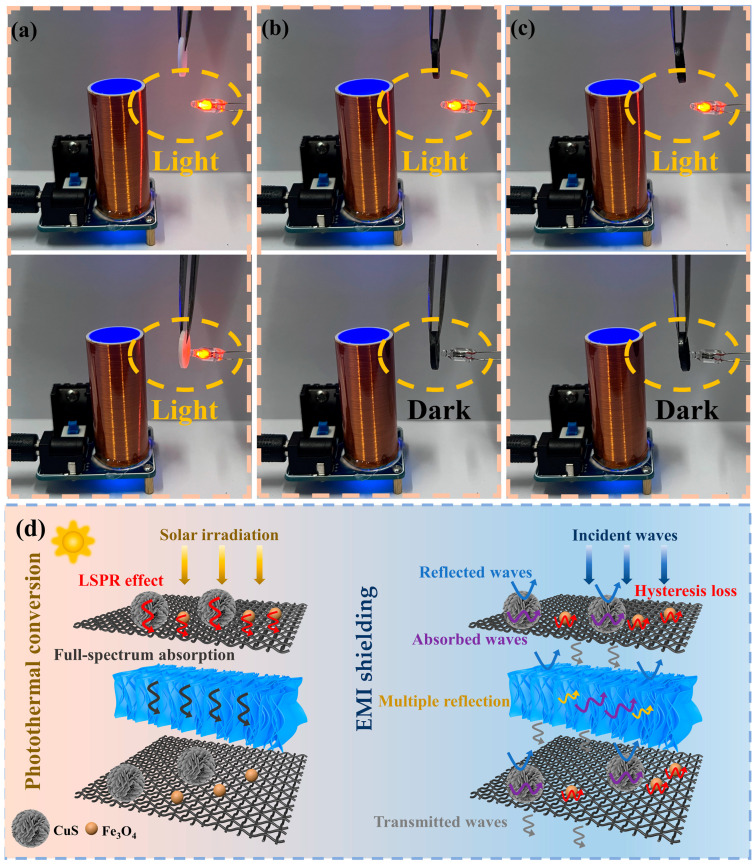
Digital photographs of Tesla kit experiment for (**a**) pure OD, (**b**) OD/3%DMDBS/6%EG, and (**c**) C-OD/3%DMDBS/6%EG; (**d**) the illustration of photothermal conversion and EMI shielding mechanisms.

The efficient and broadband EMI shielding performance of multifunctional SSPCMs will contribute to expanding its application scenarios. Next, a visual experiment is utilized to show that the prepared composite PCMs guarantee an efficacious EMI SE for actual usage [65]. Figure 11a displays a representative Tesla kit.

When the power is turned on and the switch is flipped, the DC power is converted to AC power under the action of the transistor is achieved, generating an electromagnetic field around the coil. An LED light near the coil is ignited by the electric potential created by EM inductance. When pure OD is inserted between the coil and the LED, the LED remains light (Figure 11a and Video S1), indicating that the EMI shielding effectiveness of pure OD is low. LEDs turn off when the OD/3%DMDBS/6%EG and C-OD/3%DMDBS/6%EG samples are interposed concerning coil and LEDs as EM transmission is prevented due to the excellent EMI shielding properties of these samples (Figure 11b,c and Videos S1 and S2).

With the purpose of explaining the EMI shielding mechanism visually in detail, Figure 11d illustrates the whole process of electromagnetic wave interaction with the C-OD/DMDBS/EG sandwich construction. When EM waves are incurred onto the C/CuS/ Fe_3_O_4_ interface, the EM waves interact with the Fe_3_O_4_ magnetic nanoparticles in the multifunctional interface material and generate hysteresis loss [66]. When EM waves encounter CuS loaded on cotton-derived carbon, the accumulation of radical electrons at the conducting network surface causes an impedance mismatch, leading to partial reflections. Simultaneously, the EM waves interact with abundant charge carriers, leading to substantial ohmic losses, and the resulting current reduces the energy of the EM waves, causing them to be absorbed [67]. In addition, the side length of the holes formed by the cotton-derived carbon warp and weft threads is about 500 μm, much smaller compared to the wavelengths of the EM waves in the test frequency range, which will effectively prevent the direct passage of EM waves [68]. When electromagnetic waves pass through the multifunctional interface and encounter the highly conductive OD/DMDBS/EG surface, they are immediately reflected to the multifunctional interface, and hysteresis loss and the absorption of EM waves will occur again [69]. Meanwhile, a favorable electric conducting property increases the conductive loss of EM waves. EM waves continue to pass through the OD/DMDBS/EG, which are reflected and scattered several times between the EG and within the micropore [70]. After passing through the OD/DMDBS/EG composites, the multifunctional interface shields the remaining EM waves. In conclusion, because of the synergistic enhancement of the multifunctional interfaces and the high conductivity of OD/DMDBS/EG, few EM waves transmit through the C-OD/DMDBS/EG composites. The SSPCMs with multifunctional interfaces exhibit excellent EMI shielding effectiveness.

## 4. Conclusions

In this work, we designed a multifunctional interface material. It was introduced into the PCM by the hot-press process, and a novel SSPCM with high-efficiency photothermal conversion and EMI shielding was successfully prepared. The organic/inorganic three-dimensional framework composed of DMDBS and EG was achieved to encapsulate the OD. The multifunctional interface material synergistically enhanced the OD/DMDBS/EG composite material for high solar energy utilization and EMI shielding efficiency. The main conclusions are as follows:

1. The chemical structure and microscopic morphology show that two nanoparticles with significant size differences, CuS and Fe_3_O_4_, were uniformly loaded onto the cotton-derived carbon. The PCM OD was only physically bonded to the modified material.
2. C-OD/DMDBS/EG exhibits favorable thermal storage and thermoregulation properties. C-OD/3%DMDBS/6%EG has a superior thermal storage density (200.6 J/g) and an improved thermal conductivity of 0.953 W∙m^−1^∙K^−1^. In addition, its available temperature range is extended.
3. The multifunctional SSPCMs provide highly effective photothermal conversion and EMI shielding. The photothermal conversion efficiency of C-OD/3%DMDBS/6%EG composites reaches 94.4% under 850 mW/cm^2^ illumination. In the X-band, the EMI SE averages 68.9 dB (3 mm).
4. After 300 test cycles, the ΔH_m_ and ΔH_c_ of the C-OD/3%DMDBS/6%EG composites only decrease by 2.0% and 2.2%, respectively. Moreover, the composites also show good stability after photothermal cycling tests, as well as prolonged high-temperature solar irradiation and humid environments.

In conclusion, the prepared multifunctional SSPCMs have tremendous potential for solar energy storage and electromagnetic shielding. This convenient and modular design approach also provides a new idea to achieve more efficient multifunctional drives, such as magneto-thermal, electro-thermal, and acousto-thermal, as well as the multifunctional integration of PCMs.

## Figures and Tables

**Table 1:** Peak temperatures and enthalpies of phase transition and degree of crystallinity for pure OD, OD/6%EG, OD/3%DMDBS/6%EG, and four C-OD/DMDBS/EG with different DMDBS contents.

Samples	T (°C)		ΔH (J/g)			χ_c_
	Melting	Crystallization	Melting	Crystallization	Theoretical Value	
OD	62.1	52.7	245.6	229.9	245.6	100%
OD/6%EG	61.4	52.7	220.0	196.9	230.9	95.3%
C-OD/1%DMDBS/6%EG	60.5	52.2	210.6	182.2	223.4	94.3%
OD/3%DMDBS/6%EG	58.2	51.7	204.8	178.4	223.5	91.6%
C-OD/3%DMDBS/6%EG	58.3	51.6	200.6	174.9	218.7	91.7%
C-OD/5%DMDBS/6%EG	57.9	51.0	184.5	165.2	213.7	86.3%
C-OD/7%DMDBS/6%EG	57.6	50.5	176.3	152.9	208.8	84.4%

**Table 2:** Comparison of multifunctional SSPCMs in this study and recent studies.

Multifunctional Composite PCMs	Latent Heat Storage Density (J/g)	Photothermal Conversion Efficiency (%)	EMI Shielding Effectiveness (dB)	Reference
loofah sponge/Fe_3_O_4_/paraffin wax	139.1	84	52	[59]
polyvinylidene fluoride/activated carbon/polyethylene glycol	121.3	89.42	59.83	[60]
biological porous carbon/Fe_3_O_4_/paraffin	155.2	76	32	[61]
poly (3,4-ethylene dioxythiophene) polystyrene sulfonate/MXene/polyethyleneglycol	237.6	94.9	29.8	[62]
F-reduced graphene oxide/paraffin	156.6	81.6	74.6	[63]
Hexamethylene diisocyanate trimer @ polyethyleneglycol/MXene	134	**−**	54.1	[64]
C-OD/3%DMDBS/6%EG	200.6	94.4	68.9	This work

## Data Availability

The data in this manuscript are available on request.

## Supplementary Materials

The following supporting information can be downloaded at: https://www.mdpi.com/article/10.3390/ma17040961/s1, Figure S1: The preparation process diagram of the C/CuS/Fe_3_O_4_ interface and C-OD/DMDBS/EG composites.; Figure S2: XRD patterns of (a) CuS, (b) cotton-derived carbon and (c) DMDBS.; Figure S3: FTIR spectra of cotton yarn.; Figure S4: Digital photographs of (a) cotton yarn and (b) cotton-derived carbon.; Figure S5: Relationship between gel-sol transition temperature and DMDBS content in OD/DMDBS composites.; Figure S6: Apparatus for testing the photothermal conversion performance.; Video S1: Video of Tesla kit experiment for pure OD.; Video S2: Video of Tesla kit experiment for OD/3%DMDBS/6%EG.; Video S3: Video of Tesla kit experiment for C-OD/3%DMDBS/6%EG.

## References

[1] Alawad S.M., Mansour R.B., Al-Sulaiman F.A., Rehman S. (2023). Renewable energy systems for water desalination applications: A comprehensive review. Energy Convers. Manag..

[2] Kumar A., Bhattacharya T., Mukherjee S., Sarkar B. (2022). A perspective on biochar for repairing damages in the soil–plant system caused by climate change-driven extreme weather events. Biochar.

[3] Kebede A.A., Kalogiannis T., Van Mierlo J., Berecibar M. (2022). A comprehensive review of stationary energy storage devices for large scale renewable energy sources grid integration. Renew. Sustain. Energy Rev..

[4] Sikiru S., Oladosu T.L., Amosa T.I., Kolawole S.Y., Soleimani H. (2022). Recent advances and impact of phase change materials on solar energy: A comprehensive review. J. Energy Storage.

[5] Rathore P.K.S., Gupta N.K., Yadav D., Shukla S.K., Kaul S. (2022). Thermal performance of the building envelope integrated with phase change material for thermal energy storage: An updated review. Sustain. Cities Soc..

[6] Jung Y., Kim M., Kim T., Ahn J., Lee J., Ko S.H. (2023). Functional materials and innovative strategies for wearable thermal management applications. Nano-Micro Lett..

[7] Pielichowska K., Paprota N., Pielichowski K. (2023). Fire retardant phase change materials—Recent developments and future perspectives. Materials.

[8] Dhar M., Das A., Parbat D., Manna U. (2022). Designing a network of crystalline polymers for a scalable, nonfluorinated, healable and amphiphobic solid slippery interface. Angew. Chem. Int. Ed..

[9] Li R., Zhao L., Yao A., Li Z., Wu F., Ding X., An H., Ye H., Zhang Y., Li H. (2023). A paraffin-wax-infused porous membrane with thermo-responsive properties for fouling-release microfiltration. J. Membr. Sci..

[10] Lawag R.A., Ali H.M. (2022). Phase change materials for thermal management and energy storage: A review. J. Energy Storage.

[11] Shoeibi S., Kargarsharifabad H., Mirjalily S.A.A., Sadi M., Arabkoohsar A. (2022). A comprehensive review of nano-enhanced phase change materials on solar energy applications. J. Energy Storage.

[12] Babu Sanker S., Baby R. (2022). Phase change material based thermal management of lithium ion batteries: A review on thermal performance of various thermal conductivity enhancers. J. Energy Storage.

[13] Atinafu D.G., Wi S., Yun B.Y., Kim S. (2021). Engineering biochar with multiwalled carbon nanotube for efficient phase change material encapsulation and thermal energy storage. Energy.

[14] Chibani A., Merouani S., Benmoussa F., Abdellattif M.H., Erto A., Jeon B.-H., Benguerba Y. (2021). A strategy for enhancing heat transfer in phase change material-based latent thermal energy storage unit via nano-oxides addition: A study applied to a shell-and-tube heat exchanger. J. Environ. Chem. Eng..

[15] Ghasemi K., Tasnim S., Mahmud S. (2022). Pcm, nano/microencapsulation and slurries: A review of fundamentals, categories, fabrication, numerical models and applications. Sustain. Energy Technol. Assess..

[16] Chinnasamy V., Heo J., Jung S., Lee H., Cho H. (2023). Shape stabilized phase change materials based on different support structures for thermal energy storage applications—A review. Energy.

[17] Paramparambath S., Maurya M.R., Houkan M.T., Cabibihan J.-J., Sadasivuni K.K. (2022). Improvement of heat sink performance using paraffin/graphite/hydrogel phase change composite coating. Case Stud. Therm. Eng..

[18] Ahmed S.F., Rafa N., Mehnaz T., Ahmed B., Islam N., Mofijur M., Hoang A.T., Shafiullah G.M. (2022). Integration of phase change materials in improving the performance of heating, cooling, and clean energy storage systems: An overview. J. Clean. Prod..

[19] Albdour S.A., Haddad Z., Sharaf O.Z., Alazzam A., Abu-Nada E. (2022). Micro/nano-encapsulated phase-change materials (epcms) for solar photothermal absorption and storage: Fundamentals, recent advances, and future directions. Prog. Energy Combust. Sci..

[20] Zheng X., Gao X., Huang Z., Li Z., Fang Y., Zhang Z. (2021). Form-stable paraffin/graphene aerogel/copper foam composite phase change material for solar energy conversion and storage. Sol. Energy Mater. Sol. Cells.

[21] Nishad S., Kasak P., Krupa I. (2023). Highly conductive phase change composites based on paraffin-infiltrated graphite panels for photo/electrothermal conversion and storage. J. Energy Storage.

[22] Ye X., Ma Y., Tian Z., Sun H., Zhu Z., Li J., Liang W., Li A. (2022). Shape-stable mxene/sodium alginate/carbon nanotubes hybrid phase change material composites for efficient solar energy conversion and storage. Compos. Sci. Technol..

[23] Kong L., Wang Z., Kong X., Wang L., Ji Z., Wang X., Zhang X. (2021). Large-scale fabrication of form-stable phase change nanotube composite for photothermal/electrothermal energy conversion and storage. ACS Appl. Mater. Interfaces.

[24] Atinafu D.G., Kim Y.U., Kim S., Kang Y., Kim S. (2023). Advances in biocarbon and soft material assembly for enthalpy storage: Fundamentals, mechanisms, and multimodal applications. Small.

[25] Saeed R.M.Y., Bano Z., Sun J., Wang F., Ullah N., Wang Q. (2018). Cus-functionalized cellulose based aerogel as biocatalyst for removal of organic dye. J. Appl. Polym. Sci..

[26] Adebayo L.L., Soleimani H., Yahya N., Abbas Z., Wahaab F.A., Ayinla R.T., Ali H. (2020). Recent advances in the development of fe3o4-based microwave absorbing materials. Ceram. Int..

[27] Liu L., Liang S., Cheng X., Guo M., Cheng F., Zhang M. (2022). Preparation and characterization of novel cus/sio2@n-octadecane phase-change nanocapsules enhanced photothermal conversion for solar energy utilization. Int. J. Energy Res..

[28] Dong Y., Liu H., Zhang N., Zhou J., Pan X. (2022). Photo-to-thermal conversion and energy storage of polyethylene glycol/copper sulfide composite pcms. Sol. Energy Mater. Sol. Cells.

[29] Wang L., Huang Y., Li L., Li Y., Cheng X. (2023). Binary nitrate molten salt magnetic microcapsules modified with fe3o4-functionalized carbon nanotubes for accelerating thermal energy storage. J. Energy Storage.

[30] Jannah W.N., Taufiq A., Zulaikah S., Hidayat A., Suharyadi E., Wicaksono S.T., Sunaryono S. (2023). Fe3o4–graphene/polyethylene glycol–sio2 as a phase change material for thermal energy storage. Mater. Chem. Phys..

[31] Xu J., Cheng X., Li Y., Yu G. (2019). Preparation and properties of l-octadecanol/1,3:2,4-di-(3,4-dimethyl) benzylidene sorbitol/expanded graphite form-stable composite phase change material. J. Wuhan Univ. Technol. Mater. Sci. Ed..

[32] Ren J., Wang C., Zhang X., Carey T., Chen K., Yin Y., Torrisi F. (2017). Environmentally-friendly conductive cotton fabric as flexible strain sensor based on hot press reduced graphene oxide. Carbon.

[33] Fjellvag H., Gronvold F., Stolen S. (1988). Low-temperature structural distortion in CuS. Z. Kristallogr..

[34] Radaelli P.G., Attfield J.P., Wright J.P. (2002). Charge ordered structure of magnetite Fe3O4 below the Verwey transition. Phys. Rev. B Condens. Matter. Mater. Phys..

[35] Ghosh K., Srivastava S.K. (2021). Enhanced supercapacitor performance and electromagnetic interference shielding effectiveness of cus quantum dots grown on reduced graphene oxide sheets. ACS Omega.

[36] Talebi H., Olad A., Nosrati R. (2022). Fe3o4/pani nanocomposite core-shell structure in epoxy resin matrix for the application as electromagnetic waves absorber. Prog. Org. Coat..

[37] Zhang Y., Zhu Y., Wang Z., Peng H., Yang X., Cao Y., Du C., Ma X., Cao C. (2021). Pulverization-tolerant cuse nanoflakes with high (110) planar orientation for high-performance magnesium storage. Adv. Funct. Mater..

[38] Dhiman N., Sharma V., Ghosh S. (2023). Perspective on biomass-based cotton-derived nanocarbon for multifunctional energy storage and harvesting applications. ACS Appl. Electron. Mater..

[39] Abreu A.A., Talabi S.I., de Almeida Lucas A. (2021). Influence of nucleating agents on morphology and properties of injection-molded polypropylene. Polym. Adv. Technol..

[40] Kryeziu A., Slovák V., Parchaňská A. (2022). Liquefaction of cellulose for production of advanced porous carbon materials. Polymers.

[41] Mehrose, Javed M., Qamar M.A., Shariq M., Ahmed I.A., Alziyadi K.B., Almutib E., Alaghaz A.-N.M.A., Azooz R.E., Ali S.K. (2023). Highly-efficient ni@cus/sgcn nanocomposite with superior bifunctional electrocatalytic activity for water splitting. J. Electrochem. Soc..

[42] Hosseini S.S., Hamadi A., Foroutan R., Peighambardoust S.J., Ramavandi B. (2022). Decontamination of cd2+ and pb2+ from aqueous solution using a magnetic nanocomposite of eggshell/starch/fe3o4. J. Water Process. Eng..

[43] Wang X., Wang Q., Cheng X., Chen X., Bai M. (2023). Double carbon networks reinforce the thermal storage and thermal transfer properties of 1-octadecanol phase change materials. Materials.

[44] Shimizu K., Abe F., Kishi Y., Kita R., Shinyashiki N., Yagihara S. (2023). Dielectric study on supramolecular gels by fiber structure formation from low-molecular-weight gelator/water mixtures. Gels.

[45] Nguyen G.T., Do M.H., Ly T.N., Park I., Bui T.H. (2022). Novel shape-stabilized phase change materials: Insights into the thermal energy storage of 1-octadecanol/fumed silica composites. J. Energy Storage.

[46] Gandolfo F.G., Bot A., Flöter E. (2003). Phase diagram of mixtures of stearic acid and stearyl alcohol. Thermochim. Acta.

[47] Iwasa M., Kakinoki S., Emoto K., Yoshida H. (2015). Morphology and phase transitions of n-alkyl alcohol microcrystals. J. Therm. Anal. Calorim..

[48] Chen X., Gao H., Xing L., Dong W., Li A., Cheng P., Liu P., Wang G. (2019). Nanoconfinement effects of n-doped hierarchical carbon on thermal behaviors of organic phase change materials. Energy Storage Mater..

[49] Nguyen G.T., Ly T.N., Tran N.T., Tuan H.N.A., Hieu N.H., Bui T.H. (2023). Glutaric acid/expanded graphite composites as highly efficient shape-stabilized phase change materials at medium-temperature. J. Energy Storage.

[50] Xu J., Li Y., Cheng X. (2023). Investigating the phase transition kinetics of 1-octadecanol/sorbitol derivative/expanded graphite composite phase change material with isoconversional and multivariate non-linear regression methods. Materials.

[51] Nishad S., Mohammed H., Sobolciak P., Krupa I. (2023). Evaluation of photothermal conversion performance of shape-stabilized phase change materials using a heat flux evolution curve. J. Mater. Res. Technol..

[52] Sakamoto M., Hada M., Ota W., Uesugi F., Sato T. (2023). Localised surface plasmon resonance inducing cooperative jahn–teller effect for crystal phase-change in a nanocrystal. Nat. Commun..

[53] Wahyuni S., Riswan M., Adrianto N., Dharmawan M.Y., Tumbelaka R.M., Cuana R., Istiqomah N.I., Jiananda A., Garcia S., Suharyadi E. (2023). Localized surface plasmon resonance properties dependence of green-synthesized fe3o4/ag composite nanoparticles on ag concentration and an electric field for biosensor application. Photonics Nanostruct. Fundam. Appl..

[54] Burgos J., Mondragón R., Martínez-Cuenca R., Nithiyanantham U., Barison S., Mancin S., Fabregat-Santiago F., Hernández L. (2023). Photothermal properties and performance of hybrid carbon-paraffin/water emulsions. J. Energy Storage.

[55] Ahmed A., Sharma S., Adak B., Hossain M.M., LaChance A.M., Mukhopadhyay S., Sun L. (2022). Two-dimensional mxenes: New frontier of wearable and flexible electronics. InfoMat.

[56] Oraby H., Tantawy H.R., Correa-Duarte M.A., Darwish M., Elsaidy A., Naeem I., Senna M.H. (2022). Tuning electro-magnetic interference shielding efficiency of customized polyurethane composite foams taking advantage of rgo/fe3o4 hybrid nanocomposites. Nanomaterials.

[57] Kim T., Pak S., Lim J., Hwang J.S., Park K.-H., Kim B.-S., Cha S. (2022). Electromagnetic interference shielding with 2d copper sulfide. ACS Appl. Mater. Interfaces.

[58] Kuila C., Maji A., Murmu N.C., Kuila T., Srivastava S.K. (2023). Recent advancements in carbonaceous nanomaterials for multifunctional broadband electromagnetic interference shielding and wearable devices. Carbon.

[59] He H., Dong M., Wang Q., Zhang J., Feng Q., Wei Q., Cai Y. (2023). A multifunctional carbon-base phase change composite inspired by “fruit growth”. Carbon.

[60] Fang H., Zeng J., Shao X., Hu D. (2023). Advanced electromagnetic shielding and excellent thermal management of flexible phase change composite films. Carbon.

[61] He H., Wang Y., Zhao Z., Wang Q., Wei Q., Cai Y. (2022). Dual-encapsulated multifunctional phase change composites based on biological porous carbon for efficient energy storage and conversion, thermal management, and electromagnetic interference shielding. J. Energy Storage.

[62] Jin X., Yang Z., Huang C., Yang J., Wang Y. (2022). Pedot:Pss/mxene/peg composites with remarkable thermal management performance and excellent hf-band & x-band electromagnetic interference shielding efficiency for electronic packaging. Chem. Eng. J..

[63] Li Y., Li Y., Hu W., Wang D. (2023). Shaped photothermal conversion phase-change materials with excellent electromagnetic shielding performance and flame retardancy. Adv. Eng. Mater..

[64] Fang Y., Li Z., Li X., Wu H., Sheng M., Lu X., Qu J. (2023). A novel covalent polymerized phase change composite with integrated shape memory, self-healing, electromagnetic shielding and multi-drive thermal management functions. Chem. Eng. J..

[65] Zhou M., Wang J., Zhao Y., Wang G., Gu W., Ji G. (2021). Hierarchically porous wood-derived carbon scaffold embedded phase change materials for integrated thermal energy management, electromagnetic interference shielding and multifunctional application. Carbon.

[66] Bheema R.K., Etika K.C. (2023). Large microwave absorption by fe3o4@cunw hybrid nanoparticles filled epoxy nanocomposites in the x-band. J. Alloys Compd..

[67] Yu F., Jia P., Song L., Hu Y., Wang B., Wu R. (2023). Multifunctional fabrics based on copper sulfide with excellent electromagnetic interference shielding performance for medical electronics and physical therapy. Chem. Eng. J..

[68] Guan H., Chung D.D.L. (2019). Effect of the planar coil and linear arrangements of continuous carbon fiber tow on the electromagnetic interference shielding effectiveness, with comparison of carbon fibers with and without nickel coating. Carbon.

[69] Zhang Y., Ruan K., Gu J. (2021). Flexible sandwich-structured electromagnetic interference shielding nanocomposite films with excellent thermal conductivities. Small.

[70] Guo Y., Qiu H., Ruan K., Zhang Y., Gu J. (2021). Hierarchically multifunctional polyimide composite films with strongly enhanced thermal conductivity. Nano-Micro Lett..

