# Supplementary material for: A Novel Sandwich-Structured Phase Change Composite with Efficient Photothermal Conversion and Electromagnetic Interference Shielding Interface
Source: Materials (Basel). 2024 Feb 19;17(4):961. doi: 10.3390/ma17040961 (PMC10890597; doi:10.3390/ma17040961)
Supplement: Supplementary file 1 [file materials-17-00961-s001.zip › Supplementary Information.docx]

***Electronic Supplementary Information***

A Novel Sandwich Structured Phase Change Composites with
Efficient Photothermal Conversion and Electromagnetic
Interference Shielding Interface

Jun Xu ^1^, Yuanyuan Li ^1^, Zhangxinyu Zhou ^1^ and Xiaomin Cheng ^1,2,^*

^1^ School of Materials Science and Engineering, Wuhan University of Technology, Wuhan 430070, China;

xujun22@whut.edu.cn (J.X.); yyli@whut.edu.cn (Y.L.); zzxykeyan@whut.edu.cn (Z.Z.)

^2^ School of Electromechanical and Intelligent Manufacturing, Huanggang Normal University, Huanggang 438000, China

***** Correspondence: chengxm@whut.edu.cn; Tel.: +86-135-0711-7513


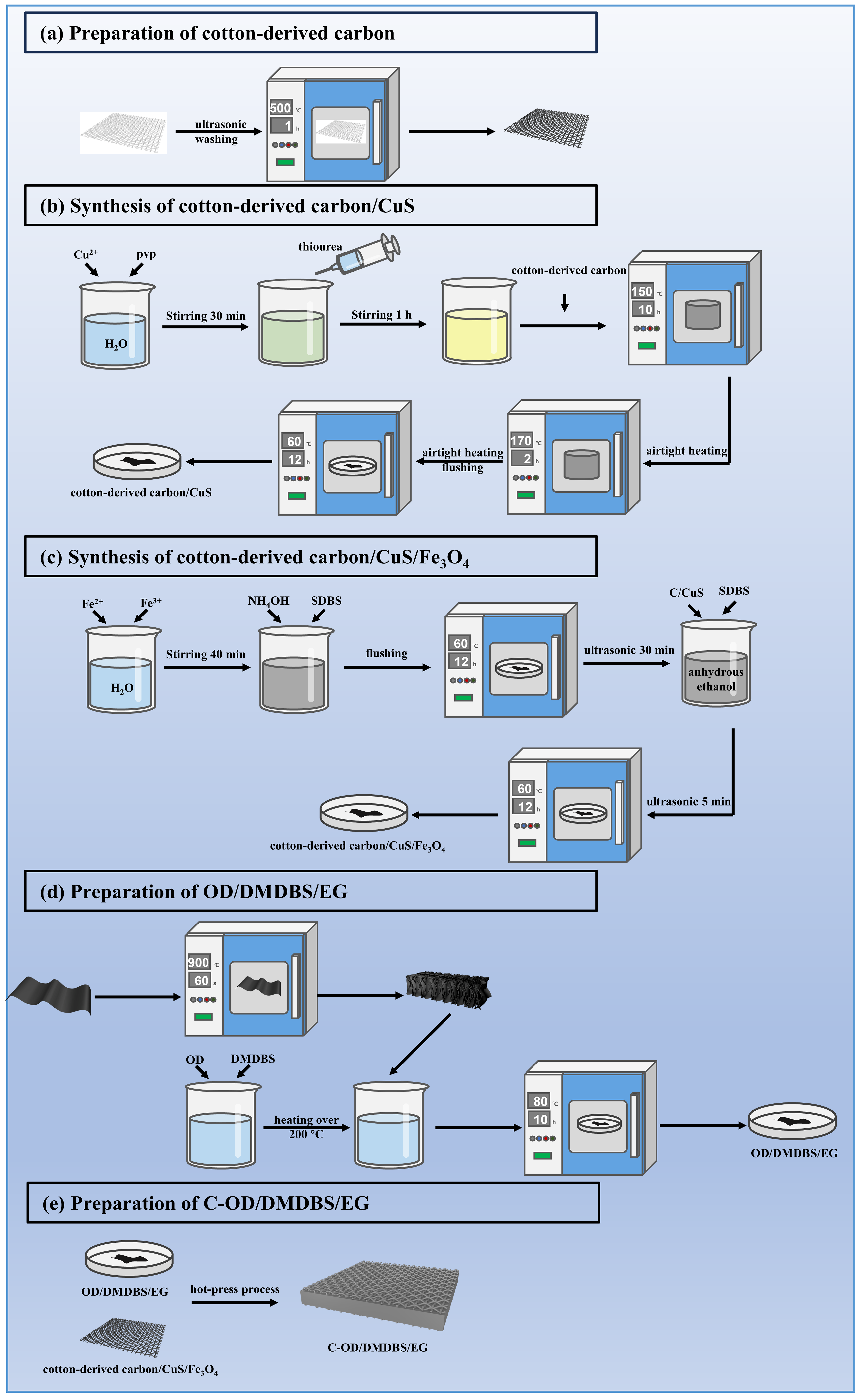


**Figure S1.** The preparation process diagram of the C/CuS/Fe_3_O_4_ interface and C-OD/DMDBS/EG composites.

S1. Materials and Methods

S1.1. Synthesis of Fe_3_O_4_ nanoparticles

FeCl_2_·4H_2_O and FeCl_3_·6H_2_O were mixed into deionised water in the ratio of 1:2.7 with stirring for 40 minutes at 80 °C. Ammonia was then added to regulate the pH value to about 10. Under alkaline conditions, Fe^2+^ and Fe^3+^ formed Fe_3_O_4_. During the preparation, SDBS was added to augment the electro-negativity of Fe_3_O_4_ and to avoid massive agglomerations. The product was collected magnetically, flushed with anhydrous ethanol and dried well to get Fe_3_O_4_ particles.

S1.2. Preparation of OD/DMDBS/EG composites

Expandable graphite (EG) was prepared via heat treatment of expandable graphite at 900 °C for 60 seconds, followed by natural cooling and subsequent dry storage. A certain proportion of OD and DMDBS was stirred continuously above 200°C until completely liquefied. EG was subsequently added and constantly stirred until complete adsorption. Eventually, the product was cooled naturally and subjected to vacuum conditions at 80 °C for 10 hours to form the OD/DMDBS/EG composites.

S1.3. Characterization

The microstructure and elemental distribution of the samples were observed by a field emission scanning electron microscopy (FE-SEM, S-4800, Japan) with an X-MAX N80 energy spectrometer and a transmission electron microscopy (TEM, Tecnai F30, Netherlands) with an energy-dispersive X-ray (EDX, Tecnai F30, Netherlands) spectroscopy. The chemical structure of the samples was characterized by an X-ray diffractometer (XRD, Panalytical-Empyrean, Netherlands) and a Fourier transform infrared (FTIR) spectrometer (Nicolet 6700, USA) at a frequency range of 4000–400 cm^−1^. A differential scanning calorimeter (DSC, NETZSCH 3500, Germany) was used to study the phase transition process of the samples at a heating and cooling rate of 10 °C/min in a nitrogen atmosphere. The samples were subjected to derivative thermogravimetric analysis (TGA-DTG) using a simultaneous thermal analyzer (STA 449 F3, NETZSCH, Germany) in a nitrogen atmosphere at a heating rate of 10 °C/min over a temperature range from room temperature to 500 °C. The thermal diffusion coefficients of samples were tested at room temperature using a laser thermal diffuser (LFA 457, NETZSCH, Germany) and the thermal conductivity was calculated using the equation (1) as follows:

| $\alpha=\frac{\lambda}{\rho\cdot C_{p}}$ | (1) |  |
| --- | --- | --- |

where λ denotes the thermal conductivity, α denotes the thermal diffusivity, ρ denotes the density, and C_p_ denotes the specific heat capacity of the samples. The light absorption properties of the samples were determined using UV-Vis spectrophotometry (Lambda 750 S, USA). The electrical conductivity was tested with a digital multimeter (HIOKI DT4215, HIOKI Electric Co., Ltd., Japan), and the electrical conductivity can be calculated by the equation (2) below:

| $\sigma=\frac{L}{S\cdot R}$ | (2) |
| --- | --- |

where σ denotes the electrical conductivity, R denotes the electrical resistance, while L and S denote the length and cross-sectional area of the samples. The electromagnetic shielding effectiveness of the samples was tested using a vector network analyzer (VNA, Agilent ENA E5071C, American) in 8.2−12.4 GHz (X-Band) using the waveguide method. The dimensions of the sample were 22.9 mm × 10.2 mm × 3.0 mm. The measured scattering parameters (S_11_ and S_21_) are used to calculate the electromagnetic shielding effectiveness using the equations (3-6) as follows：

| $R=\vert S_{11}\vert^{2}=\vert S_{22}\vert^{2},\text{ }T=\vert S_{21}\vert^{2}=\vert S_{12}\vert^{2}$ | (3) |
| --- | --- |

| $SE_{R}=-10\log(1-R)$ | (4) |
| --- | --- |

| $SE_{A}=-10\log(\frac{T}{1-R})$ | (5) |
| --- | --- |

| $SE_{T}=SE_{R}+SE_{A}$ | (6) |
| --- | --- |

where R and T denote the reflection and transmission coefficients, respectively, while SE_T_, SE_R_ and SE_A_ denote the total shielding, microwave reflection, and microwave absorption effectiveness, respectively.


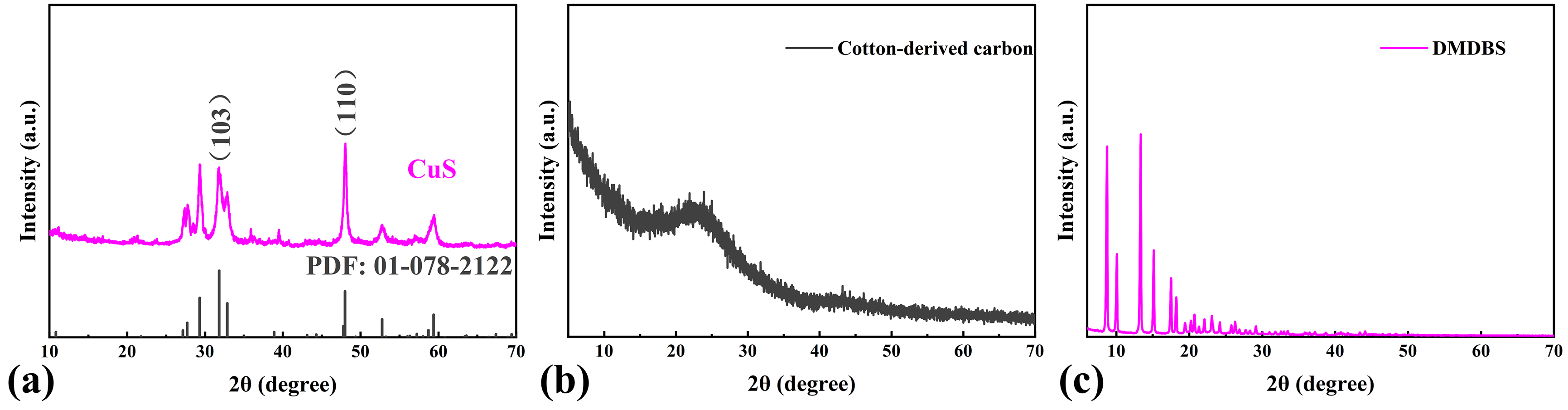


**Figure S2.** XRD patterns of (a) CuS, (b) cotton-derived carbon and (c) DMDBS.


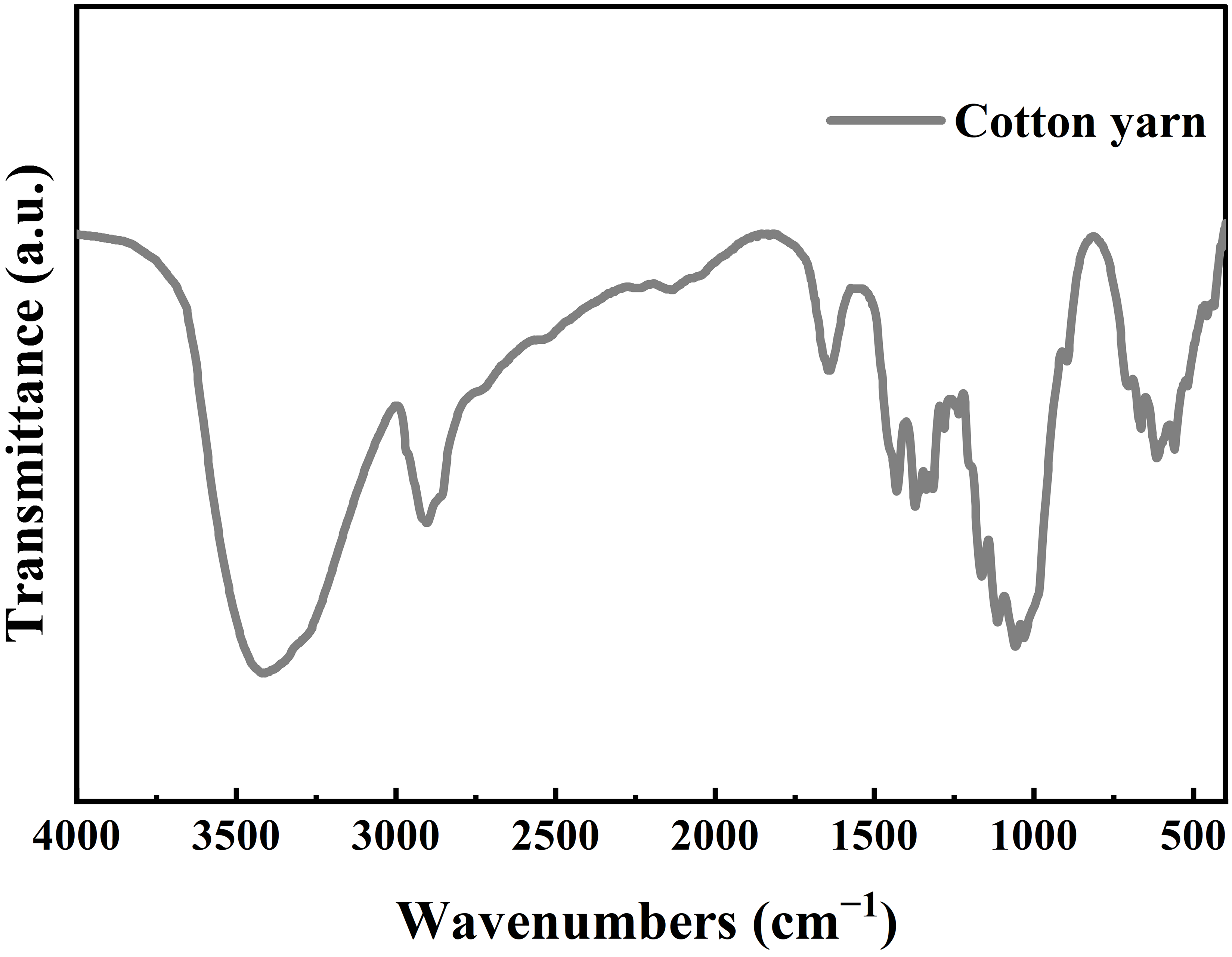


**Figure S3.** FTIR spectra of cotton yarn.


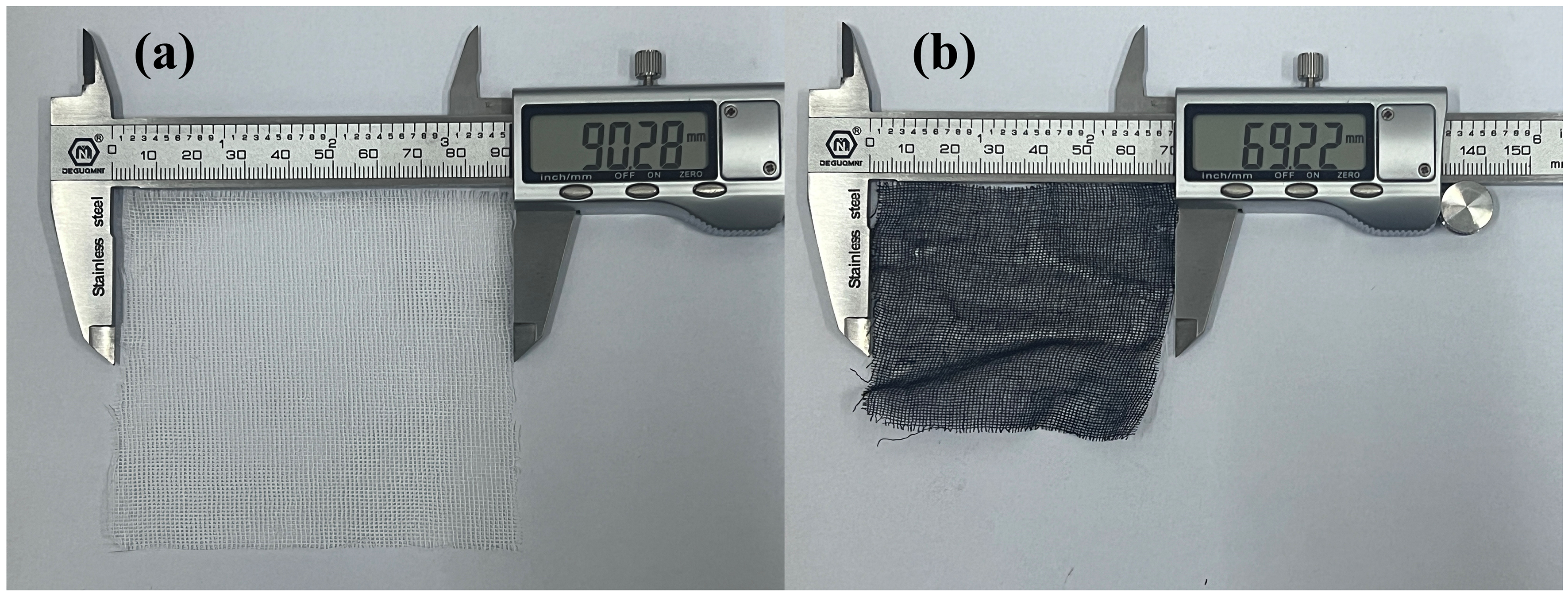


**Figure S4.** Digital photographs of (a) cotton yarn and (b) cotton-derived carbon.


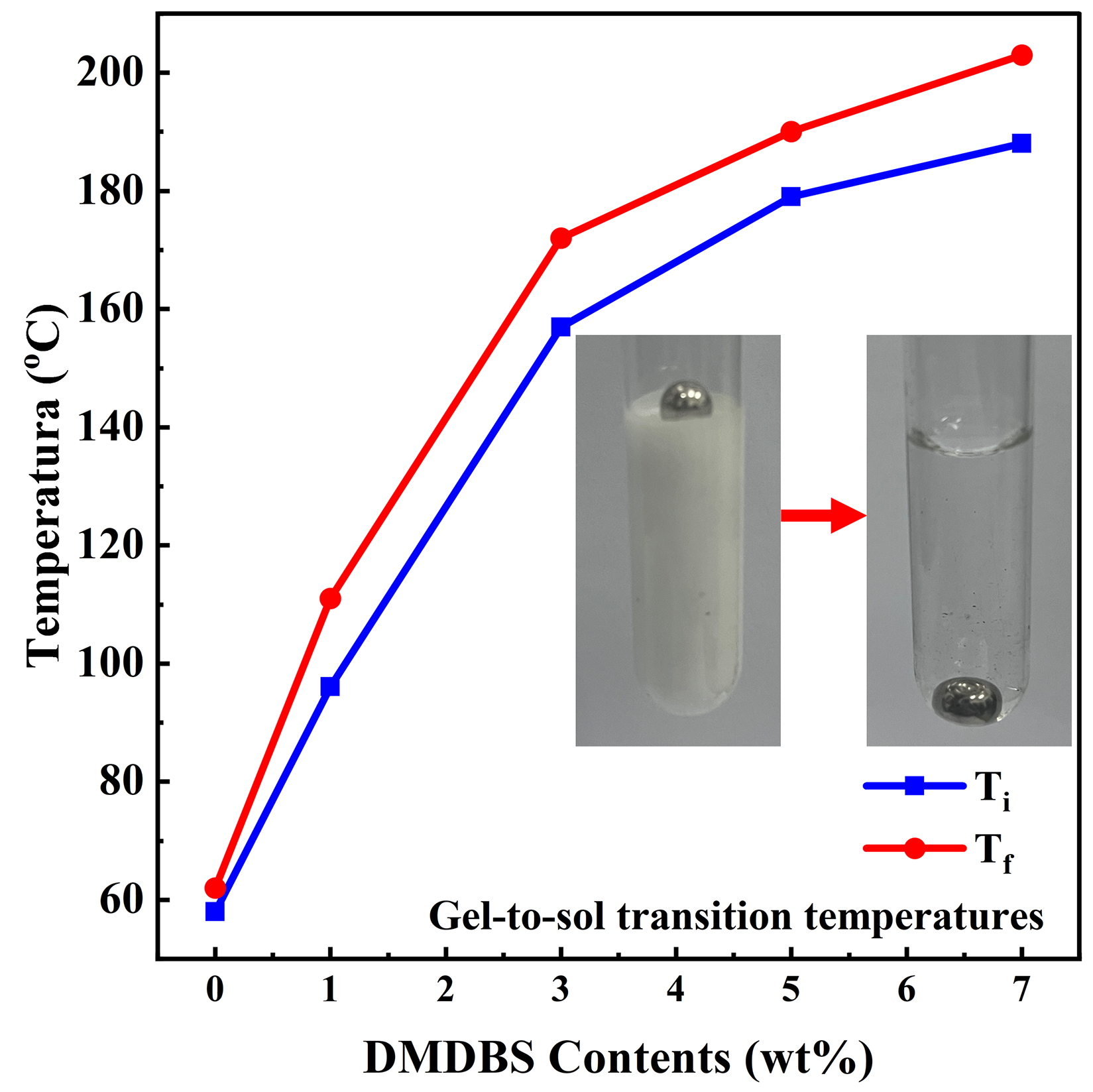


**Figure S5.** Relationship between gel-sol transition temperature and DMDBS content in OD/DMDBS composites.

The falling-ball method: A steel ball with a diameter of 5 mm was placed in a test tube containing OD/DMDBS gelatinous composites, heated in an oil bath (the heating rate was about 2 °C/min), and the oil temperature was recorded. Ts is the temperature at which the ball was just immersed into the composite, namely, the start temperature of the gel-sol transition. Te is the temperature at which the ball fell to the bottom of the test tube, namely, the end temperature of the gel-sol transition. The results are shown in Figure S5.


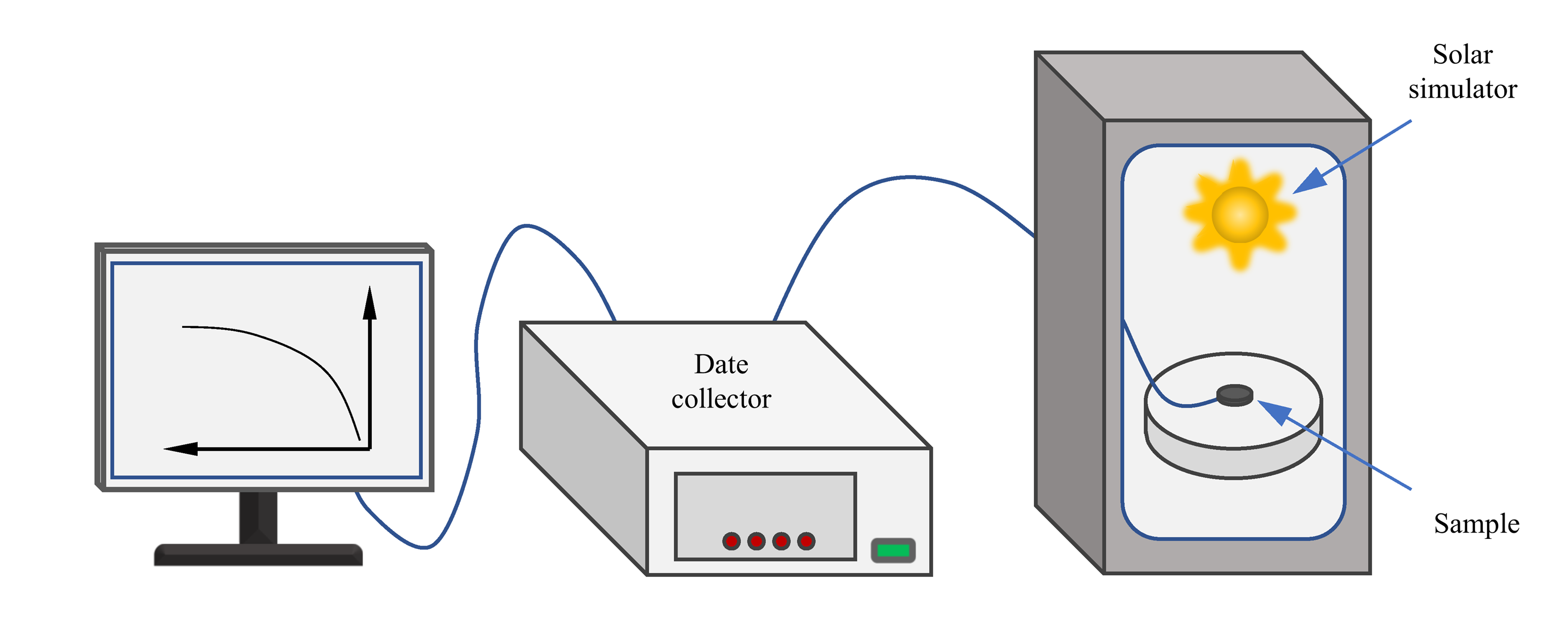


**Figure S6.** Apparatus for testing the photothermal conversion performance.

The photothermal conversion performance of the samples was measured using a self-built photothermal conversion test system, as shown in Figure S4. The system consists of a thermal insulation system (most of the thermal radiation is insulated by tin foil), a xenon lamp (CEL-HXF300-T3, China Education Au-light, China) to simulate sunlight, a full-spectrum intense light power meter (CEL-NP2000, China Education Au-light, China), a T-type thermocouple (OMEGA, SA1XL-T, USA) for temperature measurement and a multi-channel data collector (DAQ970A, Keysight, China) for data acquisition.
